# DgcA, a diguanylate cyclase from *Xanthomonas oryzae* pv. *oryzae* regulates bacterial pathogenicity on rice

**DOI:** 10.1038/srep25978

**Published:** 2016-05-19

**Authors:** Jianmei Su, Xia Zou, Liangbo Huang, Tenglong Bai, Shu Liu, Meng Yuan, Shan-Ho Chou, Ya-Wen He, Haihong Wang, Jin He

**Affiliations:** 1 State Key Laboratory of Agricultural Microbiology, College of Life Science and Technology, Huazhong Agricultural University, Wuhan, Hubei 430070, China; 2 National Key Laboratory of Crop Genetic Improvement, College of Life Science and Technology, Huazhong Agricultural University, Wuhan, Hubei 430070, China; 3 Institute of Biochemistry, and NCHU Agricultural Biotechnology Center, National Chung Hsing University, Taichung 40227, Taiwan; 4 State Key Laboratory of Microbial Metabolism, School of Life Sciences and Biotechnology, Shanghai Jiao Tong University, Shanghai 200240, China; 5 College of Life Sciences, South China Agricultural University, Guangzhou, Guangdong 510650, China

## Abstract

*Xanthomonas oryzae* pv. *oryzae* (*Xoo*) is the causal agent of rice blight disease as well as a serious phytopathogen worldwide. It is also one of the model organisms for studying bacteria-plant interactions. Current progress in bacterial signal transduction pathways has identified cyclic di-GMP as a major second messenger molecule in controlling *Xanthomonas* pathogenicity. However, it still remains largely unclear how c-di-GMP regulates the secretion of bacterial virulence factors in *Xoo*. In this study, we focused on the important roles played by DgcA (XOO3988), one of our previously identified diguanylate cyclases in *Xoo*, through further investigating the phenotypes of several *dgcA*-related mutants, namely, the *dgcA*-knockout mutant Δ*dgcA*, the *dgcA* overexpression strain O*dgcA*, the *dgcA* complemented strain C*dgcA* and the wild-type strain. The results showed that *dgcA* negatively affected virulence, EPS production, bacterial autoaggregation and motility, but positively triggered biofilm formation *via* modulating the intracellular c-di-GMP levels. RNA-seq data further identified 349 differentially expressed genes controlled by DgcA, providing a foundation for a more solid understanding of the signal transduction pathways in *Xoo*. Collectively, the present study highlights DgcA as a major regulator of *Xoo* virulence, and can serve as a potential target for preventing rice blight diseases.

*Xanthomonas oryzae* pv. *oryzae* (*Xoo*) belongs to the gamma subdivision of Gram-negative proteobacteria with a single polar flagellum^1^,^2^. It is also a serious phytopathogen causing one of the most devastating diseases in rice—bacterial leaf blight^1^,^3^, which has been estimated to incur as much as 50% economic loss of the annual rice yields worldwide^4^. Therefore, it is important to investigate the pathogenic mechanism caused by *Xoo. Xoo* enters into its host directly *via* wounds or hydathodes around leaf edges. It then multiplies either in the intercellular spaces or xylem vessels, leading to systemic spreads throughout the whole plant^3^,^4^,^5^. Typical symptoms like turning pale yellow and wilt on the leaves appear at the tillering stage after infection. The symptoms enhance slowly during plant growth, peaking at the flowering stage^4^.

Cyclic di-GMP [(bis-(3′–5′)-cyclic di-guanosine monophosphate)] is a ubiquitous second messenger that has been found to extensively involve in regulating a wide range of bacterial physiological functions^6^,^7^. It is synthesized from two molecules of GTP by diguanylate cyclases (DGCs) containing the GGDEF domain, and hydrolyzed by phosphodiesterases (PDEs) containing either an EAL or HD-GYP domain to generate linear dinucleotide pGpG or guanosine monophosphate (GMP), respectively^7^. These three domains are broadly distributed among microbes and are commonly associated with various signal-sensing domains such as PAS, GAF, REC and BLUE to sense a plethora of environmental cues^6^,^7^. Intracellular c-di-GMP then binds with a wide variety of receptors to control the corresponding biological processes, including bacterial motility, aggregative behavior, adhesion, biofilm formation, cell division, and virulence^6^,^7^. Therefore, characterization of proteins that alter c-di-GMP levels temporarily or spatially is of crucial importance to understand how this second messenger modulates such diverse biological functions^8^.

The genus *Xanthomonas* comprises 27 species, with quite a few of DGCs and PDEs predicted in the genomes of *Xcc* 8004^9^,^10^,^11^, *Xoc* BLS256^12^ and *Xoo* PXO99^A ^ strains^13^. Recently, our group identified approximately 8–17 GGDEF domain-containing proteins, 10–13 EAL domain-containing proteins, and 1–3 HD-GYP domain-containing proteins among the 15 full-sequenced *Xanthomonas* genomes by bioinformatics analyses^14^. To our knowledge, none of these DGCs in *Xoo* has been intensively investigated to date, and only one c-di-GMP metabolic protein PdeR (PXO_01019)^15^ containing a dual GGDEF-EAL domain has been found to function as a PDE in *Xoo*. Deletion of PdeR resulted in a substantial reduction of virulence factor productions in *Xoo*^15^.

One of the major pathways controlling pathogenic networks in *Xoo* is the type II secretion system (T2SS), which regulates the secretion of various virulence factors, including extracellular polymeric substances (EPS) xanthan and extracellular enzymes (such as protease, amylase, endoglucanase, cellulase, lipase, pectinase, aminopeptidase, mannanase and polygalacturonate lyase, to name a few) to outside of the cell that collectively contribute to the *Xoo* virulence^11^,^16^,^17^. Similar to other Gram-negative phytopathogenic bacteria, *Xoo* also depends on a highly conserved type III secretion system (T3SS) to translocate T3 effectors into the host cells^18^,^19^. Notably, different rice cultivars exhibit different degrees of pathogenicities because some of the rice plants like Zhonghua 11 may carry the elicitor-specific resistance genes that confer some degree of resistance to rice blast^18^,^19^,^20^.

In order to further investigate the signal transduction regulated by c-di-GMP in *Xoo*, a predicted DGC gene *dgcA* (XOO3988, also called XOO_RS19705) from *Xoo* KACC10331 (*Xoo* 10331) was cloned and verified to function as an active DGC *in vitro* and *in vivo*^21^. In the present study, we further constructed the *dgcA*-knockout mutant Δ*dgcA*, the *dgcA* overexpression strain O*dgcA*, and the *dgcA* complemented strain C*dgcA* to investigate its function in a more detailed way. The obtained results demonstrated that *dgcA* did play a crucial role in controlling the *Xoo* pathogenicity, and deletion of *dgcA* led to significant increases in virulence, EPS production, autoaggregation activity as well as swimming motility, but to a drastic reduction in biofilm formation. RNA-seq technology further identified a wide range of differentially expressed genes (DEGs) between the Δ*dgcA* mutant with WT. Such data provided valuable information for understanding the mechanisms involved in triggering virulence and different phenotypes of *Xoo*. Together, our findings strongly suggest that the c-di-GMP level in *Xoo* is significantly affected by *dgcA*, which seems to serve as a major regulator in controlling bacterial signal transduction and pathogenicity in *Xoo*.

## Results

### DgcA contains an intact GGDEF domain that functions as an active DGC

Bioinformatic analysis reveals that there are eight genes encoding GGDEF domain-containing proteins in the *Xoo* 10331 genome (NC_006834.1) (Supplementary Fig. S1a), with many of them annotated as “hypothetical proteins”. XOO3988, annotated as a “response regulator”, contains a typical GGDEF domain and was previously designated as DgcA^21^. In addition, DgcA also possesses a HAMP (histidine kinases, adenylyl cyclases, methyl-binding proteins and phosphatases) domain, which is presumed to play a central role in signal transduction. Protein sequence of DgcA displays a high (92%) identity with XCC3486^22^ that also contains a HAMP-GGDEF domain. Although the crystal structure (PDB: 3QYY) of XCC3486^22^ has been solved, its biological function remains unknown (Supplementary Fig. S1b).

TMHMM2 software predicted DgcA containing two TM regions at the N-terminus (Supplementary Fig. S1a). A truncate tDgcA protein without the TM domain (tDgcA) was thus constructed by attaching a His-tag at the C-terminus. The tDgcA was successfully expressed and purified to a final concentration of 1.8 mg ml^−1^, as shown by a single band of 26.4 kDa in SDS-PAGE (Supplementary Fig. S2a). This truncated tDgcA containing the GGDEF domain was found to be active in converting GTP to c-di-GMP *in vitro* as checked by HPLC analysis (Supplementary Fig. S2b). Moreover, it was also found to exhibit significant DGC activity *in vivo* by using our previously constructed dual-fluorescence reporter system mediated by a triple-tandem (Bc3–5 RNA) riboswitch^21^, which can monitor the activity of a putative DGC in a highly sensitive and effective way *in vivo*.

### DgcA boosts the intracellular c-di-GMP level of *Xoo*

**Figure 1:**
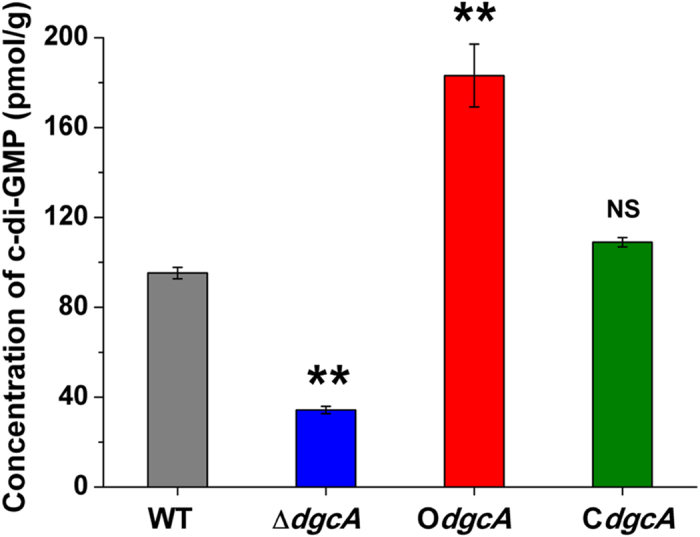
Intracellular concentrations of c-di-GMP in WT, Δ*dgcA*, O*dgcA* and C*dgcA* strains determined by LC-MS/MS. The values were means ± standard deviations for triplicate assays. Significances of differences by Student’s *t*-test are indicated (**P < 0.01; *P < 0.05; NS, P > 0.05).

*Xoo* mutant strians Δ*dgcA*, O*dgcA* and C*dgcA* were confirmed by PCR using the corresponding primers (Supplementary Fig. S3b,c), followed by sequencing. Western blotting analysis showed an apparent band with a molecular weight of 35.5 kDa in the O*dgcA* and C*dgcA* strains (Supplementary Fig. S3d). The mean intracellular c-di-GMP concentration in O*dgcA* reached to 183.1 pmol g^−1^, which was higher than that in C*dgcA* (109.0 pmol g^−1^) (Fig. 1). In contrast, the mean intracellular concentration of c-di-GMP in Δ*dgcA* was only 34.3 pmol g^−1^, which was 2.8-fold lower than that of WT (95.3 pmol g^−1^) (Fig. 1). In a word, these results clearly indicate that DgcA functions as a main DGC *in vivo* to alter the intracellular level of c-di-GMP in *Xoo*.

### Deletion of *dgcA* substantially increases *Xoo* virulence against two different rice cultivars

**Figure 2:**
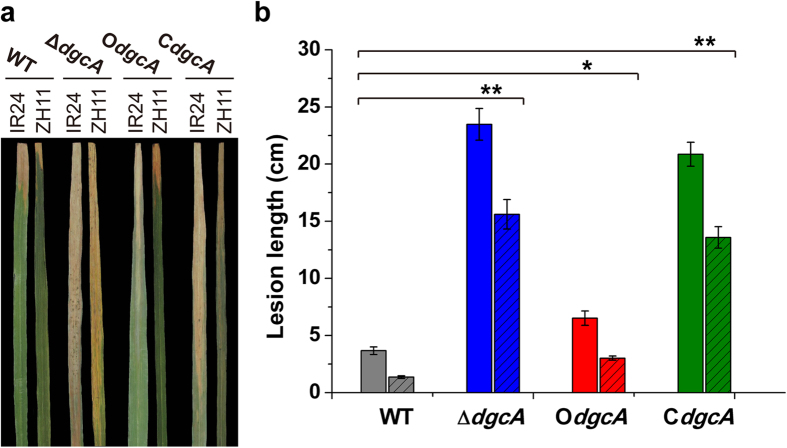
*In Planta* virulence test of WT, Δ*dgcA*, O*dgcA* and C*dgcA* strains. (**a**) Leaf segments of rice cultivars IR24 and Zhonghua 11 (ZH11) showing different degree of rice blight symptoms. Typical leaves were photographed after infection with the four individual strains for 14 days. (**b**) The mean lesion of IR24 (left column) and Zhonghua 11 (right column with slash) lengths 14 days post inoculation. Leaf without bacterial inoculation were performed as a negative control. The values were means ± standard deviations for triplicate assays. Significances of differences by Student’s *t*-test are indicated (**P < 0.01; *P < 0.05; NS, P > 0.05).

After inoculation for 14 days, the blight lesions caused by the four *Xoo* strains could be obviously observed on the rice leaves (Fig. 2a). While leaves inoculated with WT *Xoo* exhibited an average lesion length of 4 cm for susceptible IR24 cultivar and 1 cm for resistant Zhonghua 11 cultivar, respectively (Fig. 2b), those inoculated by Δ*dgcA* resulted in a substantial lesion increase to an average length of 24 cm in IR24, and an average length of 16 cm in Zhonghua 11. The complementary C*dgcA* strain, on the other hand, did not completely decrease its virulence towards the WT level, but only alleviated approximately 10–15% of virulence on the blight symptoms (see Discussion below) (Fig. 2a,b). Unexpectedly, overexpression of *dgcA* did not reduce the virulence infection compared with WT but instead only moderately enhanced the average lesion lengths to 6 cm and 3 cm for the IR24 and Zhonghua 11 cultivars (Fig. 2b), respectively. Considering that higher lesion length might be caused by the introduction of the overexpressing plasmid, we determined the pathogenicity of Xoo-pBBR strain containing an empty vector pBBR1MCS-2 as a control. The result showed that the empty vector control exhibited no effect on the *Xoo* virulence, and the Xoo-pBBR strain displayed a similar lesion length on Zhonghua 11 rice leaf as WT (Supplementary Fig. S4a,b). Almost no lesion was observed when rice leaf was inoculated with sterilized M210 medium.

Bacterial populations in rice leaves after inoculation for 3, 6, 9 and 12 days (Supplementary Fig. S4c) were then determined respectively. Firstly, we confirmed that bacteria isolated from the inoculated rice leaves were indeed *Xoo* strains according to their specific colony morphology and color. After infection for 9 or 12 days, it was obvious that more bacteria occurred in leaves inoculated with Δ*dgcA* and C*dgcA*. The *in planta* population size of O*dgcA* was obviously smaller than the above two strains, but still larger than those of WT and Xoo-pBBR strains (Supplementary Fig. S4c). These bacteria populations in leaves were consistent with the virulence behaviors exhibited during infection.

### DgcA slightly affects the bacterial growth

**Figure 3:**
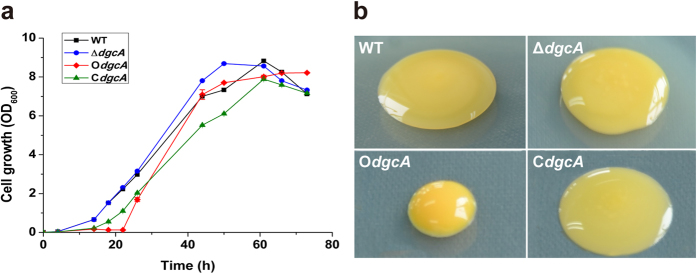
Bacterial growth phenotypes of WT, Δ*dgcA*, O*dgcA* and C*dgcA* strains in the M210 medium and agar plates. (**a**) Growth curves of the four bacteria grown at 28 °C in the M210 medium. The values were means ± standard deviations for triplicate assays. (**b**) Observation of colony morphology after growing for three days at 28 °C on the M210 agar plates.

Bacterial growth curves demonstrated that O*dgcA* indeed had a longer lag phase compared with other strains, but then grew almost similarly with the WT and Δ*dgcA* strains, and even higher than C*dgcA* after entering the late logarithmic phase at 44 h (Fig. 3a). Besides, the colony of O*dgcA* exhibited a deeper yellow color and was significantly smaller in size than the other three strains grown on the same plate (Fig. 3b).

### DgcA represses EPS production, autoaggregation and motility abilities

**Figure 4:**
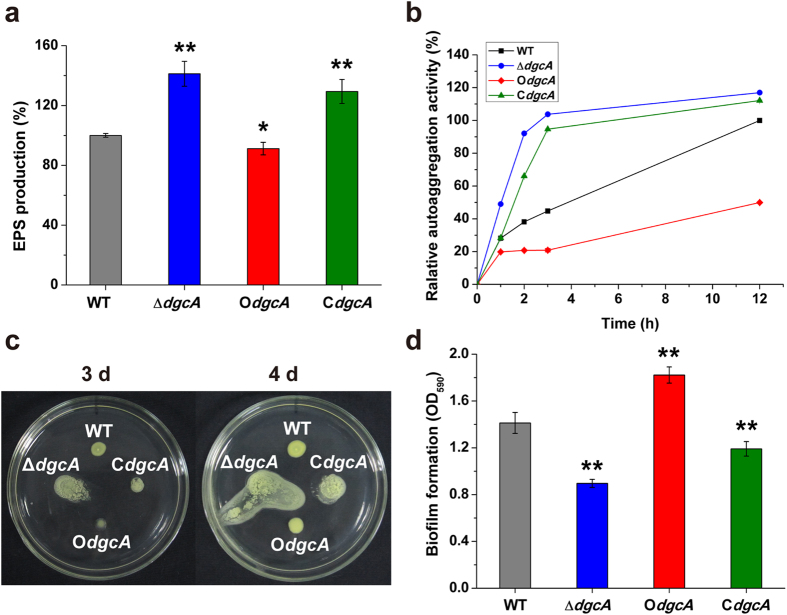
Phenotypic changes of EPS, autoaggregation, motility and biofilm in WT, Δ*dgcA*, O*dgcA* and C*dgcA* strains. (**a**) Quantitative measurement of EPS productions in the four strains after cultivated in the M210 medium for 44 h. (**b**) Determination of autoaggregation ability in these four strains after growing at 28 °C for 44 h and kept static at room temperature for 0, 1, 2, 3 and 12 h respectively. (**c**) Detection of swimming motility of the four strains on the semi-solid M210 medium plates with 0.3% agar after cultivated for three and four days. (**d**) Measurement of biofilm formation by crystal violet staining. Biofilms were grown in glass tubes for five days at 28 °C under static conditions. The values were means ± standard deviations for triplicate assays. Significances of differences by Student’s *t*-test are indicated (**P < 0.01; *P < 0.05; NS, P > 0.05).

As shown in Fig. 4a, while O*dgcA* generated only a lower amount of EPS than WT by 8.8%, the Δ*dgcA* and C*dgcA* strains displayed a 41.1% and 29.1% increase in EPS production compared to that of WT, respectively. The result suggested that DgcA inhibited the production of EPS, which could only be partially restored in the complementary C*dgcA* strain.

In order to investigate whether DgcA regulated the autoaggregation phenotype, a sedimentation assay was further carried out. After sitting statically on the bench for 12 h, the cells of Δ*dgcA* and C*dgcA* rapidly sank to the tube bottom and formed large precipitates, which displayed 117.0% and 112.2% of sedimentation percentages compared with WT (100%), respectively (Fig. 4b). However, overexpression of *dgcA* resulted in the dispersion of culture and a muddy appearing supernatant. The O*dgcA* strain showed only 50.0% of autoaggregation after 12 h, which was 2 times less than WT, indicating that DgcA impaired the sedimentation process and weakened the autoaggregative phenotype.

In the swimming motility assay, Δ*dgcA* was found to form a large diffuse colony surface on the M210 medium after three days, which was in sharp contrast to the small swimming diameter observed in the WT and C*dgcA* strains (Fig. 4c). When *dgcA* was overexpressed in O*dgcA*, the strain turned sticky and non-motile, exhibiting a very small colony diameter with almost no migration after three days (Fig. 4c). However, this swimming-defective phenomenon was alleviated on the fourth day as the colony diameter of O*dgcA* quickly increased to a size similar to WT. Additionally, robust motility phenotypes were even more significant for the Δ*dgcA* and C*dgcA* strains after cultivated on the plates for four days, and it was surprising to observe that bacteria colony turned watery and secreted some matrixes around the colony (Fig. 4c). Although the level of bacterial surface migration in C*dgcA* was smaller than Δ*dgcA*, it still exhibited an increased swimming motility compared with WT, hinting that the motility ability was partially restored.

### Biofilm formation is markedly increased by DgcA

Biofilm biomass formed by Δ*dgcA* was reduced by 36.6% than that of WT, and this decrease of biofilm formation could be rescued to some extent by the complementation of *dgcA* in C*dgcA* (Fig. 4d). In contrast, an increased amount of biofilms was produced and adhered to the tube surface at the air-liquid interface in O*dgcA*, which exhibited a substantially higher level (29.0%) of biofilm biomass than WT (Fig. 4d). Thus, the ability to form biofilm was highly dependent on the DgcA activity.

### Significance of DEGs analyzed by COGs, GO and KEGG

**Figure 5:**
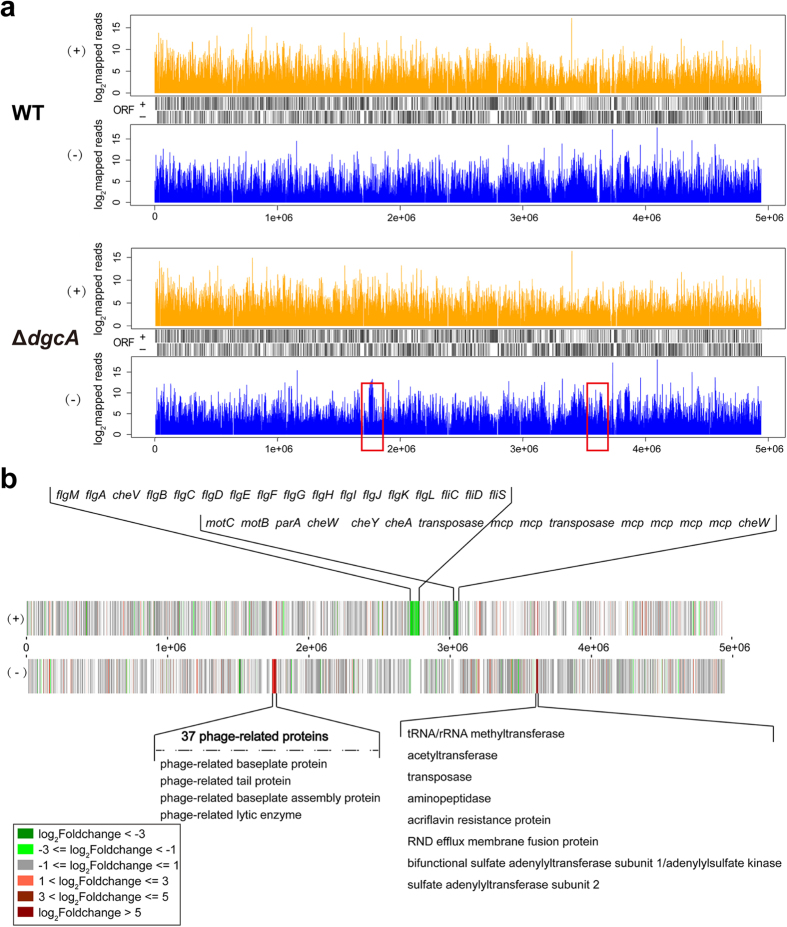
Coverage of clean reads mapped to the genomes of WT and Δ*dgcA*. (**a**) Mapping information count for each base. The red rectangular frames indicate the most significant differences between WT and Δ*dgcA*. (+) and (−) represent sense and anti-sense strands, respectively. (**b**) Heatmap of DEGs distributed in the WT genome. Color scale in the bottom left corner indicates fold changes in gene expression. Red color indicates up-regulated gene while green color indicates down-regulated gene.

To get a clearer picture about the importance of DgcA in controlling the virulence of *Xoo* in rice, we conducted a detailed study on the gene expression profiles by RNA-seq of the WT and Δ*dgcA* strains. After omitting the low quality sequence reads by PRINSEQ, the clean reads of WT and Δ*dgcA* could reach to 90–95% of the total raw reads, indicating a confidential RNA-seq data (Supplementary Table S1). The whole clean reads were successfully mapped to the *Xoo* 10331 (WT) genome using the Bowtie2 program^23^. As could be seen in Fig. 5a, the mapped reads distributed uniformly in the genome with a perfect match and coverage. In total, 349 DEGs were successfully identified by DEGSeq^24^ from Δ*dgcA*, including 190 up-regulated DEGs and 159 down-regulated DEGs, compared with WT. Notably, two major different patterns of reads were clearly identified in the RNA-seq data between the Δ*dgcA* and WT strains (indicated in boxed frames).

These DEGs on both strands were further analyzed and plotted with a visual heatmap to facilitate comparison of gene expression levels by displaying with different colors. The generated heatmap showed that many genes associated with the flagellar assembly and bacterial chemotaxis on the sense strand were dramatically down-regulated, whereas 37 phage-related genes and several genes encoding transferases (such as tRNA/ rRNA methyltransferase, acetyltransferase, transposase and so on) on the antisense strand were significantly up-regulated, respectively (Fig. 5b).

**Figure 6:**
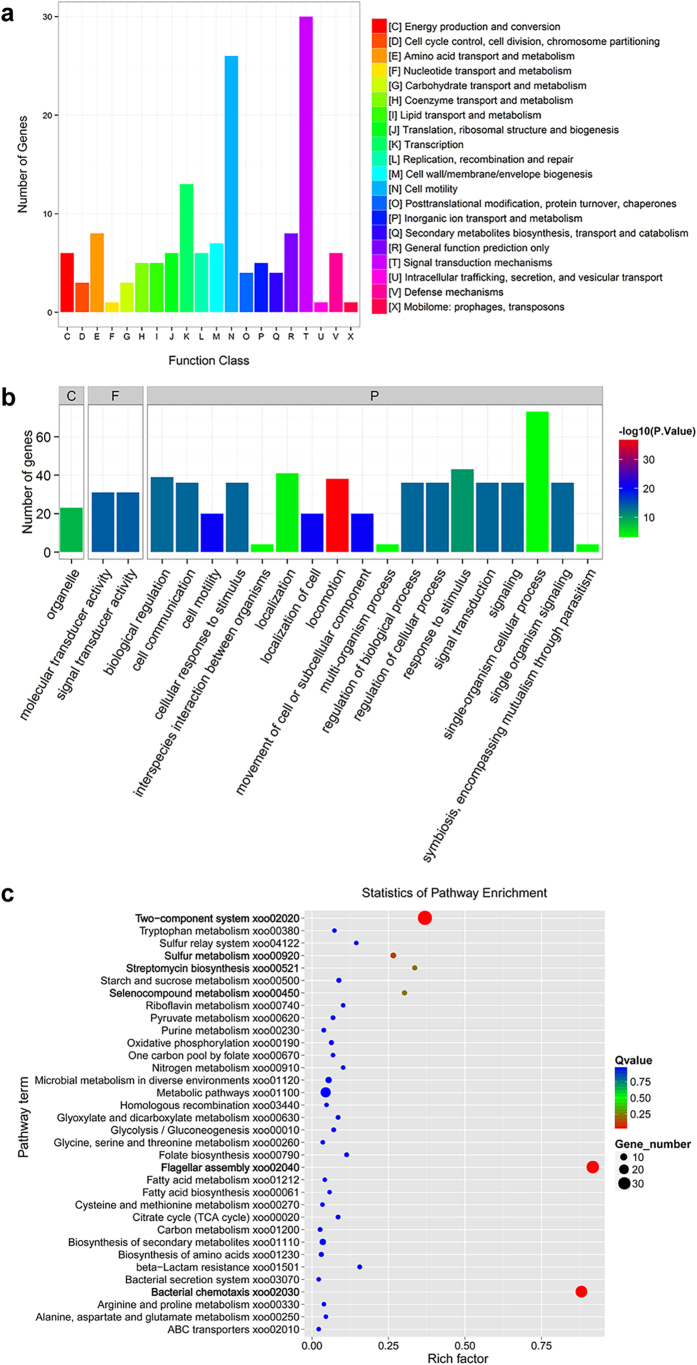
COG, GO and KEGG enrichment analyses of DEGs between WT and Δ*dgcA* strains. (**a**) Histogram presentation of COG classification. A total of 148 DEGs were successfully annotated and grouped into 20 COG functional categories. (**b**) GO classification map. 176 DEGs were annotated and divided into three categories: ‘cellular component’ (C), ‘molecular function’ (F) and ‘biological process’ (P). GO terms were sorted based on p-values. (**c**) DEGs enriched in the KEGG Pathway. Dots represent term enrichment with different colors based on Q values. Red color indicates high enrichment, while blue color indicates low enrichment. The sizes of the dots represent the gene number of enrichment.

Based on the conserved domain alignment, these 349 DEGs were searched against the Clusters of Orthologous Groups (COGs) database to predict and classify their possible functions. In total, 148 DEGs were successfully annotated and grouped into 20 COG functional categories, in which the cluster of ‘signal transduction mechanisms’ occupied the highest number (30; 20.3%), followed by ‘cell motility’ (26; 17.6%) and ‘transcription’ (13; 8.8%) (Fig. 6a).

Furthermore, DEGs were also subject to Gene ontology (GO) enrichment analysis to predict their potential biological functions. 176 DEGs were successfully annotated and were divided into three categories: ‘cellular component’ (C), ‘molecular function’ (F) and ‘biological process’ (P) (Fig. 6b). The ‘Organelle’ consisting of 23 genes was the only term enriched in the C category by Blast2GO^25^. Two GO terms ‘signal transducer activity’ and ‘molecular transducer activity’ were also identified in the F category. High percentages of genes related to ‘single-organism cellular process’, ‘response to stimulus’, ‘localization’ and ‘locomotion’ were observed to be dominantly represented in the P category. Since the majority of genes were assigned to the dominant P category, GO-Termfinder^25^ was applied to analyze significantly overrepresented terms in the P category and to display them in the context of the GO tree pipeline. The enrichment results showed that genes with respect to ‘sulfate assimilation’ and ‘viral capsid assembly’ were markedly up-regulated because 44% genes were annotated as transposases in the 190 up-regulated DEGs (Supplementary Fig. S5A). In contrast, genes correlated to ‘cell motility’ and ‘chemotaxis’ were dramatically down-regulated (Supplementary Fig. S5B).

Finally, Kyoto Encyclopedia of Genes and Genomes (KEGG) pathway enrichment analysis was employed to identify the most significantly metabolic or signal transduction pathways for all DEGs compared with the whole WT genome background. Among the 34 KEGG pathways, 184 DEGs were matched, with the most representative pathways correlated to the ‘two-component system’ (KEGG-ID: xoo02020), ‘flagellar assembly’ (KEGG-ID: xoo02040) and ‘bacterial chemotaxis’ (KEGG-ID: xoo02030), which all displayed a significantly lower Q value of <0.05 (Fig. 6c). Besides, other KEGG pathways involved in the ‘streptomycin biosynthesis’ (KEGG-ID: xoo00521), ‘sulfur metabolism’ (KEGG-ID: xoo00920), and ‘selenocompound metabolism’ (KEGG-ID: xoo00450) were also considerably enriched with a Q value of <0.25 (Fig. 6c). All these enriched pathways between Δ*dgcA* and WT indicated that they might participate in the c-di-GMP signaling networks and contribute to the phenotypic changes.

## Discussion

The importance of c-di-GMP in bacterial physiology has been well investigated to date, and a large number of DGCs containing the conserved GGDEF domain were discovered in most bacterial strains^10^,^26^. In *Xcc*, a dual GGDEF-EAL domain-containing protein Xcc_1958 (RavR, also a response regulator) and three GGDEF domain-containing proteins Xcc_2731^27^, Xcc_1294^16^ and Xcc_4471^28^ were shown to exhibit DGC activities^29^. However, relatively few DGCs were identified and studied in the rice-infecting *Xoo*; only a response regulator PXO_ 01019 (PdeR) containing a dual GGDEF-EAL domain was reported to exhibit a PDE activity in *Xoo* PXO99^A ^15. In this study, DgcA was annotated as a “response regulator” similar to that reported for the PleD DGC^26^. Since full-length DgcA and truncated tDgcA were both found to exert DGC activity *in vivo*^21^ and tDgcA could further synthesize c-di-GMP *in vitro*, the biological roles of DgcA were extensively explored in the present study by investigating three different mutant strains of Δ*dgcA*, O*dgcA* and C*dgcA*. We demonstrated in the present manuscript that DgcA served as an effective regulator in controlling biofilm formation, EPS production, autoaggregation, motility and virulence through altering intracellular c-di-GMP levels in these strains.

As expected, the intracellular c-di-GMP levels in O*dgcA* and Δ*dgcA* strains were significantly higher and lower than WT, respectively. Interestingly, two genes encoding GGDEF domain-containing proteins (XOO0111 and XOO2787) and one gene encoding GGDEF-EAL dual domain-containing protein (XOO1551) were observed to be markedly down-regulated in the Δ*dgcA* strain from RNA-seq data. Meanwhile, five genes encoding EAL-containing proteins (XOO1467, XOO2354, XOO2561, XOO2616 and XOO3246) were also found to be down-regulated (Supplementary Table S2). These data suggested that deletion of *dgcA* might affect the transcriptions of these GGDEF/EAL domain-containing proteins, but their effects on the intracellular c-di-GMP level were uncertain. The activities of GGDEF/EAL domain-containing proteins have been found to be modulated in complex manners, including allosteric regulation by GTP binding, by phosphorylation of other domains in the protein, by localization to micro-domains within the cell, as well as at the levels of transcription and protein stability^30^. Whether these GGDEF/EAL domain-containing proteins function as active DGCs and PDEs *in vivo* require further investigations by mutating each of these genes. The reduced intracellular concentration of c-di-GMP in Δ*dgcA* may be coordinately caused by the direct effect of *dgcA* deletion, and/or by the indirect effect of changed activities of other DGCs and PDEs due to *dgcA* deletion. Whether direct or indirect, the experimental data together indicate that *dgcA* does play a key role in regulating the intracellular c-di-GMP amounts in *Xoo*.

It has been well documented that a higher c-di-GMP concentration is associated with a number of characteristic phenotypes, such as promoted attachment and biofilm formation, but suppressed motility and bacterial virulence^31^,^32^,^33^. In the present study, deletion of *dgcA* was indeed found to severely impair biofilm formation, whereas overexpression of *dgcA* significantly increased biofilm formation. These results were consistent with many other previous reports^9^,^34^. Apart from biofilm formation, c-di-GMP is also known to govern the switch between motile and sessile cell phenotypes^34^. O*dgcA* with a higher c-di-GMP concentration was found to be completely non-motile on swimming plates and did not swim at all in any direction after cultivation for three days. In contrast, deletion of *dgcA* enabled Δ*dgcA* to swim freely and smoothly in the same semi-solid media. Complementation of *dgcA* in this strain did not restore the swimming phenotype back to the WT level, although the swimming motility was decreased to some extent in C*dgcA*. The incomplete restoration in C*dgcA* was probably attributed to the strict control of *dgcA* dosage within the cells, where a slight perturbation in gene copy number by a complementation plasmid might result in measurable differences in motility^35^,^36^. In fact, c-di-GMP has been found to inhibit motility *via* several different regulatory mechanisms through controlling transcription, post-transcription, translation, and assembly of flagella stages^32^,^37^. Additionally, bacterial motility including both flagellar swimming motility and twitching motility mediated by type IV pili (T4P) was also verified to contribute to bacterial virulence^38^. Considering that most flagellar genes in *Xoo* are located in a single cluster of about 40 ORFs (from XOO2565 to XOO2621), and two other flagellar genes *motC* (XOO2830) and *motB* (XOO2831) are located in a chemotaxis cluster downstream of the main flagellar cluster^35^, we therefore further analyzed the expressions of these genes in transcriptional level. Among these genes, only two pili-related genes of *pilE1* (XOO3195) and *pilY1* (XOO3196), which were required for protein secretion, bacterial aggregation, adhesion, motility, biofilm formation and virulence^35^,^38^, were strongly up-regulated according to the RNA-seq data. However, other genes associated with flagellar assembly were almost all down-regulated in Δ*dgcA* compared with WT from the RNA-seq data. One possible explanation for these transcriptional differences is that our present RNA-seq data only reflects the transcription profile at 44 h (at the late logarithmic phase), while the motility phenotype is usually recorded after cultivation in plates for three or four days at the declining phase.

Intriguingly, Δ*dgcA* and C*dgcA* strains grown on the semi-solid agar apparently secreted some unknown extruded matrixes around the bacterial colony, where the colony edge could not stop quickly enough to prevent forming an anomalous shape. Similar abnormal surface spreading phenomenon was reported previously^39^,^40^ and the extruded matrixes were considered likely to be EPS, which primarily drove the colony to expand outward on the surface from the inoculation point. The quantitative result on EPS production by Δ*dgcA* further confirmed that deletion of *dgcA* significantly increased the formation of EPS while overexpression of *dgcA*, on the contrary, remarkably reduced its formation. Additionally, the increase of autoaggregation in Δ*dgcA* cells under low c-di-GMP concentration might be due to reinforced cell-cell interactions modulated by the increased EPS or altered cell surface properties^31^. Although both EPS production and autoaggregation phenotypes in this study were decreased with increasing c-di-GMP levels, which were opposite to the common concept that higher intracellular c-di-GMP favored increased EPS formation and autoaggregation in other strains, other observations reported in *Xcc*^11^,^41^ were in line with our result that c-di-GMP negatively influenced biosynthesis of EPS xanthan.

Xanthan is possibly the major EPS produced by *Xoo* that has been proved to play a crucial role in triggering plant disease^9^,^17^. Besides EPS, the increased motility of Δ*dgcA* is also responsible for the enhanced virulence as wound inoculation enables the mutant to more rapidly access to the plant xylem vessel and bypass the hydathode over a larger area, leading to the enhanced necrosis phenotype^20^,^41^. Other virulence factors such as extracellular enzymes, adhesins, T3SS and secreted effectors are also required for full *Xoo* pathogenicity^15^. A decrease of c-di-GMP concentration generally leads to the syntheses of virulence factors and dispersal of biofilms^27^. From the transcriptome analysis, we found that many enzymes such as peptidase or aminopeptidase (belonged to T2SS) were up-regulated in Δ*dgcA*. Importantly, 37 phage-related genes and several genes encoding transferases (such as tRNA/rRNA methyltransferase, acetyltransferase, transposase and so on) were also found to be considerably up-regulated, suggesting that they might play predominant roles in intraspecific evolution by bacteriophages in the infection process^42^,^43^. Moreover, transposases even accounted for up to 44% of total up-regulated DEGs, which was probably due to the large amount of insertion sequence (IS) elements that endow the *Xoo* genome with strong plasticity and rapid adaptability. The rise in expressions of these mobility-related genes presumably contributes to their insertion and excision from chromosomes through horizontal gene transfer, which is potentially related to bacterial pathogenicity during infection process^42^. Interestingly, the Zhonghua 11 rice carrying resistance genes was less infected compared with the susceptible rice IR24, suggesting that host plant should also be considered as a factor affecting bacterial virulence^44^. Taken together, these data hinted that a lower c-di-GMP concentration promoted bacterial pathogenesis in rice plant.

Since Δ*dgcA* is a single gene, deletion of *dgcA* has no effect on the transcription of downstream gene XOO3989 (*pepN*) (Supplementary Fig. S6). Generally, the downstream cellular functions regulated by c-di-GMP often depend on signal transduction by its receptors or effectors. To date, the identified c-di-GMP receptors in genus *Xanthomonas* include transcription factor CRP-like protein (Clp)^8^, degenerate GGDEF-EAL domain proteins Filp^45^ and FimX^46^, several PilZ domain proteins such as PXO_00049, PXO_02374^33^, and XCC6012^46^, and a recently discovered YajQ family protein XC_3703^47^. In this study, we found that the homologous receptors Clp (XOO4158), a YajQ family protein XOO0711, and three PilZ domain proteins XOO0887, XOO2849 and XOO1138 were existent in *Xoo*. Among them, only XOO2849 was significantly down-regulated, which might affect the sliding motility of Δ*dgcA* as the homologous protein PXO_00049 in *Xoo* PXO99^A ^33. One probable explanation for the little expression changes of these possible receptors might be attributed to the spatial sequestration or asymmetric distribution of c-di-GMP^48^, where the levels of c-di-GMP adjacent to these possible receptors had little difference between the WT and Δ*dgcA*, suggesting that some other undiscovered receptors might be existent in *Xoo*.

A total of 349 DEGs were identified in Δ*dgcA*, including 190 up-regulated genes and 159 down-regulated genes compared to WT, suggesting that *dgcA* exhibits a very complicated and crucial role in regulating the c-di-GMP signaling network in *Xoo*. The KEGG pathway enrichment analysis showed that the highest score genes identified belonged to the ‘two-component system’ (37) pathway. Moreover, COG analysis demonstrated that 30 DEGs (20.3%) were classified into ‘signal transduction mechanisms’. Similarly, the GO clustering results found that both ‘signal transducer activity’ (31) and ‘molecular transducer activity’ (31) occupied for about 4.8% in the F category. All these results revealed that the two-component signal transduction systems (TCS), which regulate the expression of bacteriocins and various toxins, as well as the production of other proteins involved in virulence and pathogenicity^2^,^49^, were closely related to c-di-GMP regulatory network. The transcriptomic analyses conducted in this study provide a useful platform for accelerating researches on the c-di-GMP-mediated processes, on the critical functions of DEGs, and on the major metabolic pathways in *Xoo* affected by the *dgcA* gene.

In conclusion, the present study demonstrated that DgcA negatively affected virulence, EPS production, bacterial autoaggregation and motility, but positively influenced biofilm formation through modulation of c-di-GMP levels from studies of four different *Xoo* strains. Furthermore, next generation sequencing technique revealed a considerable number of DEGs regulated by c-di-GMP, which are related to *Xoo* pathogenicity. Further analyses of these enriched gene clusters or represented pathways will not only serve as a valuable resource to better understand underlying biological and physiological mechanisms of c-di-GMP-induced phenotypic changes, but also shed a new light on the identification of novel targets for the treatment of serious bacterial leaf blight disease.

## Methods

### Bacterial strains, plasmids, primers and growth conditions

*Xoo* 10331^2^ and its derivates, *Escherichia coli* strains and plasmids used in this study were listed in Table 1. All primers in the experiments were also listed in Supplementary Table S3.

*E. coli* S17-1 λpir and BL21(DE3) strains were cultivated in LB medium at 37 °C. *Xoo* strains were routinely grown in the nutrient-rich medium M210^15^ (8 g of casein enzymatic hydrolysates, 4 g l^−1^ of yeast extract, 5 g l^−1^ of sucrose, 3 g l^−1^ of K_2_HPO_4_, 0.3 g l^−1^ MgSO_4_·7H_2_O). The antibiotics kanamycin (Km, 50 μg ml^−1^), gentamicin (Gm, 30 μg ml^−1^) and cephalexin (Cep, 24 μg ml^−1^) were added when required. Rice plants (the indica cultivar IR24 and japonica cultivar Zhonghua 11)^44^ used for virulence activity test were grown in irrigated land for 60 days.

### Bioinformatics analysis

A search of the *Xoo* 10331 genome (GenBank accession NC-006834.1) for proteins containing GGDEF domains was carried out by the HMMER program. The resulting protein sequences were then subjected to Pfam (http://pfam.janelia.org/) and Smart database (v4.0, http://smart.embl-heidelberg.de/help/smart_glossary.shtml) to identify all conserved organized domains. Transmembrane domains of these proteins were predicted by the TMHMM2 program (http://www.cbs.dtu.dk/services/TMHMM/, version 2.0). Amino acid alignment using Clustal Omega (http://www.ebi.ac.uk/Tools/msa/clustalO/) was applied to verify the conserved sequences of proteins.

### DGC activity assay

Truncated tDgcA protein (10 μM) purified from BLtdgcA^21^ was added into reaction mixtures containing 100 mM Tris-HCl (pH 8.0) and 10 mM MgCl_2_ in a 100 ml volume, and the reactions were started by addition of 0.1 mM GTP. After incubated at 37 °C for 24 h, the enzymatic reaction was stopped by heating to 95 °C for 5 min and then centrifuged at 12,000 rpm for 10 min to remove denatured proteins. The obtained supernatant was then loaded onto an Elite Hypersil BDS C18 Column (200 × 4.6 mm, 5 μm particle sizes) for monitoring the formation of c-di-GMP using a Shimadzu LC-20AT HPLC instrument (mobile phase: 90% 20 mM ammonium acetate (pH 6.8) and 10% methanol; flow rate: 1 ml min^−1^). Assays were performed in triplicate.

### Constructions of gene deletion mutant, overexpressed and complemented strains of *Xoo*

In order to create the *dgcA*-knockout mutant Δ*dgcA*, a marker exchange strategy was applied using the suicide vector pK18*mobsacB via* allelic homologous recombination^3^,^50^. The construction procedure was drawn in Supplementary Fig. S3a. In brief, the upstream and downstream arms of *dgcA* were amplified from the *Xoo* genome DNA with two pairs of primers *dgcA*-up-F/*dgcA*-up-R and *dgcA*-down-F/*dgcA*-down-R (Supplementary Table S3), respectively. A Gm resistance gene for allelic exchange was amplified from plasmid pBBR1MCS-5^3^,^51^ with the primer pair Gm-F and Gm-R (Supplementary Table S3). The obtained DNA fragments were digested with corresponding restriction enzymes and cloned into pK18*mobsacB*^15^ in order, resulting in a plasmid pK18Δ*dgcA* (Table 1). After sequencing, the plasmid was conjugated from *E. coli* S17-1 λpir into *Xoo* 10331 by biparental mating as described previously^52^. Mutants were selected on the M210 plates containing Cep and Km for the first crossover event. Positive colonies were then plated on the M210 plates containing 6–10% (w/v) sucrose and 30 μg ml^−1^ Gm to screen for a second crossover event^3^. The Δ*dgcA* candidates were subsequently confirmed by PCR and DNA sequencing.

The coding region of *dgcA* was amplified with primer pair *dgcA*-F2 and *dgcA*-R2 (Supplementary Table S3), which was then ligated into plasmid pBBR1MCS-2 carrying a strong T3 promoter^51^ at the HindIII and BamHI sites. The recombinant pBBR1MCS-*dgcA* plasmid was then introduced into the competent cells of *Xoo* 10311 (WT) and Δ*dgcA* by electroporation^3^,^15^ to generate overexpression strain O*dgcA* and complemented strain C*dgcA*, respectively. The expression of *dgcA* in both strains were verified by Western blotting assay with the anti-His mouse monoclonal antibody according to the manufacturer’s instructions (TransGen Biotech Co., Ltd., Beijing, China).

### Extraction and quantification of intracellular c-di-GMP by LC-MS/MS

Each single colony of the above four strains was diluted to an initial OD_600_ of 0.05 and was inoculated into 150 ml of M210 medium containing corresponding antibiotic respectively. After growing at 28 °C, 200 r min^−1^ for 44 h, the cell were collected and centrifuged at 4 °C for 20 min at 4000 r min^−1^, and further washed twice with fresh medium. Nucleotides were extracted using a protocol as described previously^53^ and analyzed by liquid chromatography-tandem mass spectrometry (LC-MS/MS) with a Thermo Scientic TSQ Quantum Ultra EMR tandem mass spectrum system (San Jose, CA, USA)^54^. Intracellular c-di-GMP level was presented as pmol g^−1^ bacterial protein.

### Virulence assays on rice plants

The pathogenicities of WT, Δ*dgcA*, O*dgcA*, and C*dgcA* strains were investigated by the leaf-cutting method^2^. Rice lines IR24 (susceptible)^18^,^55^ and Zhonghua 11 (carring some resistance genes)^44^ were used as hosts for virulence tests. The fully expanded leaves were inoculated by clipping leaf tips with sterile scissors dipped in freshly diluted bacterial suspensions (OD_600_ = 0.8, approximately 4 × 10^9^ CFU ml^−1^)^56^. Control inoculations were performed simultaneously by immersing leafs in sterilized M210 liquid medium. At least 25 leaves were inoculated and scored for each tested *Xoo* strain. Symptoms were scored by measuring lesion lengths after 14 days of inoculation and the values are presented as mean lesion lengths ± SD^56^. The entire experiment was repeated three independent times.

For monitoring bacterial populations *in planta*, rice leaves were harvested at four specific time points (3, 6, 9, 12 days after inoculation) for each treatment. After immersed into 75% ethanol for 1 min and subsequently sucked dry with a sterile filter paper, the leaves were sliced into small pieces, and ground in 1 ml of water using a sterilized mortar and pestle^9^,^18^. Afterwards, the resulting suspensions were serially diluted and then plated in triplicate onto the M210 agar plates with antibiotics, if needed. Colonies on the plates with appropriate dilutions were counted after three days of incubation at 28 °C.

### Phenotype differences of the four strains

Four streaked strains of WT, Δ*dgcA*, O*dgcA*, and C*dgcA* were respectively picked up from plates and inoculated into the M210 medium (containing antibiotic if needed), which were then grown for three days with shaking at 28 °C. Each culture was diluted to a starting OD_600_ of approximately 0.01 in 100 ml of fresh M210 medium containing the required antibiotic, which was then incubated at 28 °C with shaking for about 44 h to reach the late logarithmic phase. This activated culture was diluted to an OD_600_ of 0.05 and prepared for the following phenotypic assays.

Growth curve was measured by taking aliquots of culture at approximately 4 h intervals until 73 h, and the OD_600_ values of each strain were recorded and plotted versus time^9^. For colony morphology observation, cultures were initiated at an OD_600_ of 0.05 and 3 μl of each culture was dropped onto the M210 plates. Cells were grown at 28 °C and monitored after three days.

After growing at 28 °C for 44 h, half cultures were collected by centrifugation at 10,000 × g for 10 min for measuring the EPS production as reported^1^,^5^,^11^. 10 ml cultures were transferred to a 15 ml round bottom glass tube to determine the autoaggregation ability as previously described^31^,^57^. These tubes were allowed to sit without agitation at room temperature for 0, 1, 2, 3 and 12 h respectively. The OD_600_ of the supernatant in the static tube culture was determined to monitor relative autoaggregation activity, which was calculated using the OD difference value relative to the densities at the initial experiment^57^.

Swimming motility assays were investigated on the semi-solid M210 medium plates with 0.3% agar as described^15^. Aliquots (3 μl) of each diluted culture were spotted onto the plates, which were photographed after incubating at 28 °C for three and four days, respectively^3^,^50^. The experiments were repeated at least three times and a representative result was chosen for display. Biofilm formation assay was carried out by incubating the diluted culture for five days at 28 °C under static conditions. Non-adherent bacteria and medium were discarded and biofilm was washed with deionized water. The attached biofilm was stained with 0.1% (w/v) crystal violet for 30 min at room temperature^58^, thoroughly washed with water for 3 times and air-dried. The bound dye was solubilized in 90% ethanol and quantified by measuring absorption at 590 nm^9^. All the phenotypic assays were performed in triplicate.

### RNA isolation and quantitative transcriptomics (RNA-seq)

The WT strain and Δ*dgcA* strain were grown in the M210 medium with appropriate antibiotics at 28 °C, and cells were then harvest at late logarithmic phase (44 h) by centrifugation at 8000 × g for 20 min at 4 °C. Total RNA was isolated using the Trizol (Invitrogen, Carlsbad, CA) method as suggested in the manufacturer’s protocol and dissolved in 40–60 μl RNase-free water. cDNA synthesis and Illumina Sequencing were performed as described^24^. Experiments were carried out in triplicate with independently prepared samples.

### RNA-Seq data analysis

The quality of the raw sequence data was first assessed by FastQC, and the sequences were then filtered and trimmed by PRINSEQ^23^. The resulting clean reads were mapped and aligned to the *Xoo* 10331 genome sequence by Bowtie2^23^. To facilitate the comparison of expression levels of different genes, data for CDSs were presented in the form of reads per kilobase per million reads (RPKM). DEGs between WT and Δ*dgcA* strain were identified by the DEGseq package with MARS (MA-plot-based method with Random Sampling model) method^24^. We simply defined the FDR (false discovery rate) less than 0.001 and log_2_ (fold-change) value more than 1.0 as criteria to screen DEGs.

To facilitate the global analysis of gene expression, genes were sorted to the different COGs according to the COG data available on the Wego website (http://wego.genomics.org.cn/)^25^. In addition, GO annotation and enrichment analyses were performed using a Blast2Go and GO-TermFinder (0.86) based on the results of blastx. Functional classification and pathway assignment of DEGs were performed by the KEGG using KOBAS software (KOBAS, Surrey, UK)^25^.

## Additional Information

**How to cite this article**: Su, J. *et al.* DgcA, a diguanylate cyclase from *Xanthomonas oryzae* pv. *oryzae* regulates bacterial pathogenicity on rice. *Sci. Rep.* **6**, 25978; doi: 10.1038/srep25978 (2016).

## Supplementary Material

Supplementary Information

## Figures and Tables

**Table 1:** Bacterial strains and plasmids used in this study.

Strains and plasmids	Descriptions	References or sources
**Strains**		
BL21(DE3)	*E. coli*; F^−^*ompT* r_B_^−^ m_B_^−^ (λDE3)	Invitrogen
S17-1 λpir	*E. coli*; Tmp^r^ Sp^r^ Sm^r^ *pro hsdR* RP4-2-Tc::Mu-::Tn7λpir	This study
*Xoo* 10331 (WT)	*Xanthomonas oryzae* pv. *oryzae* KACC10331, Cep^r^	5
Δ*dgcA*	In frame deletion of *dgcA* derived from *Xoo*, Gm^r^	This study
C*dgcA*	**∆*dgcA* containing an expression vector pBBR1MCS-*dgcA*	This study
O*dgcA*	*Xoo* containing an expression vector pBBR1MCS-*dgcA*	This study
Xoo-pBBR	*Xoo* containing an empty vector pBBR1MCS-2	This study
BLtdgcA	BL21(DE3) containing pET-28b(+)-	21
**Plasmids**		
pK18*mobsacB*	Allelic exchange vector, Km^r^, *sacB*, Tra^−^, Mob^+^	This study
pBBR1MCS-5	Gm^r^ pBBR1 broad host range derivative vector, RK2 *mob* region	33
pK18Δ*dgcA*	Kan^r^, Gm^r^; pK18*mobsacB*, Δ*dgcA* (in frame)	This study
pBBR1MCS-2	Gm^r^; hexahistidine fusion and expression vector	51
pBBR1MCS-	Kan^r^, DgcA overexpression vector in *Xoo*	This study
pET-28b(+)-*tdgcA*	Gm^r^; tDgcA overexpression vector in *E. coli*	21
