# Supplementary material for: DgcA, a diguanylate cyclase from Xanthomonas oryzae pv. oryzae regulates bacterial pathogenicity on rice
Source: Sci Rep. 2016 May 19;6:25978. doi: 10.1038/srep25978 (PMC4872155; doi:10.1038/srep25978)
Supplement: Supplementary Information [file srep25978-s1.pdf]

DgcA, a diguanylate cyclase from *Xanthomonas oryzae* pv. *oryzae* regulates bacterial pathogenicity on rice

Jianmei Su[1], Xia Zou[1], Liangbo Huang[1], Tenglong Bai[1], Shu Liu[1], Meng Yuan[2], Shan-Ho Chou[3], Ya-Wen He[4], Haihong Wang[5], Jin He[1]*.

[1]State Key Laboratory of Agricultural Microbiology, College of Life Science and Technology, Huazhong Agricultural University, Wuhan, Hubei 430070, China.

[2]National Key Laboratory of Crop Genetic Improvement, College of Life Science and Technology, Huazhong Agricultural University, Wuhan, Hubei 430070, China.

[3]Institute of Biochemistry, and NCHU Agricultural Biotechnology Center, National Chung Hsing University, Taichung 40227, Taiwan.

[4]State Key Laboratory of Microbial Metabolism, School of Life Sciences and Biotechnology, Shanghai Jiao Tong University, Shanghai 200240, China.

[5]College of Life Sciences, South China Agricultural University, Guangzhou, Guangdong 510650, China.

*Corresponding author:

E-mail: hejin@mail.hzau.edu.cn (JH)

Section A: supplementary figures

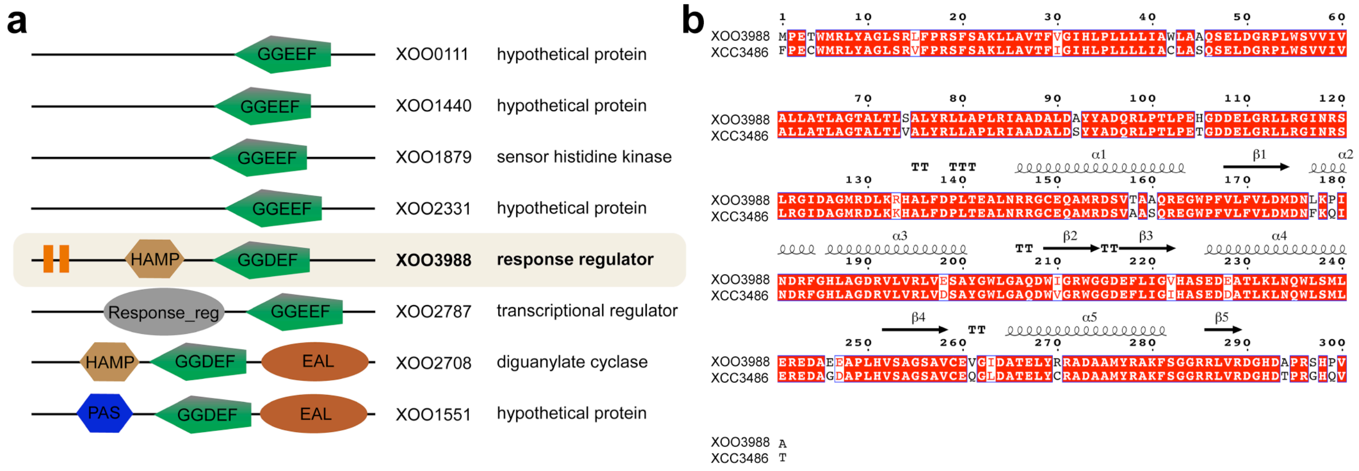

Figure S1. Multidomain architectures of DGCs and sequence alignment of DgcA with XCC3486. (a) Architectures of GGDEF domain-containing proteins with a GGDEF or GGEEF motifs in the *Xoo* 10331 genome. Predicted TM regions are shown as orange rectangles. (**b**) The amino acid sequence alignment of DgcA (XOO3988) in this study with XCC3486 from *Xcc*.

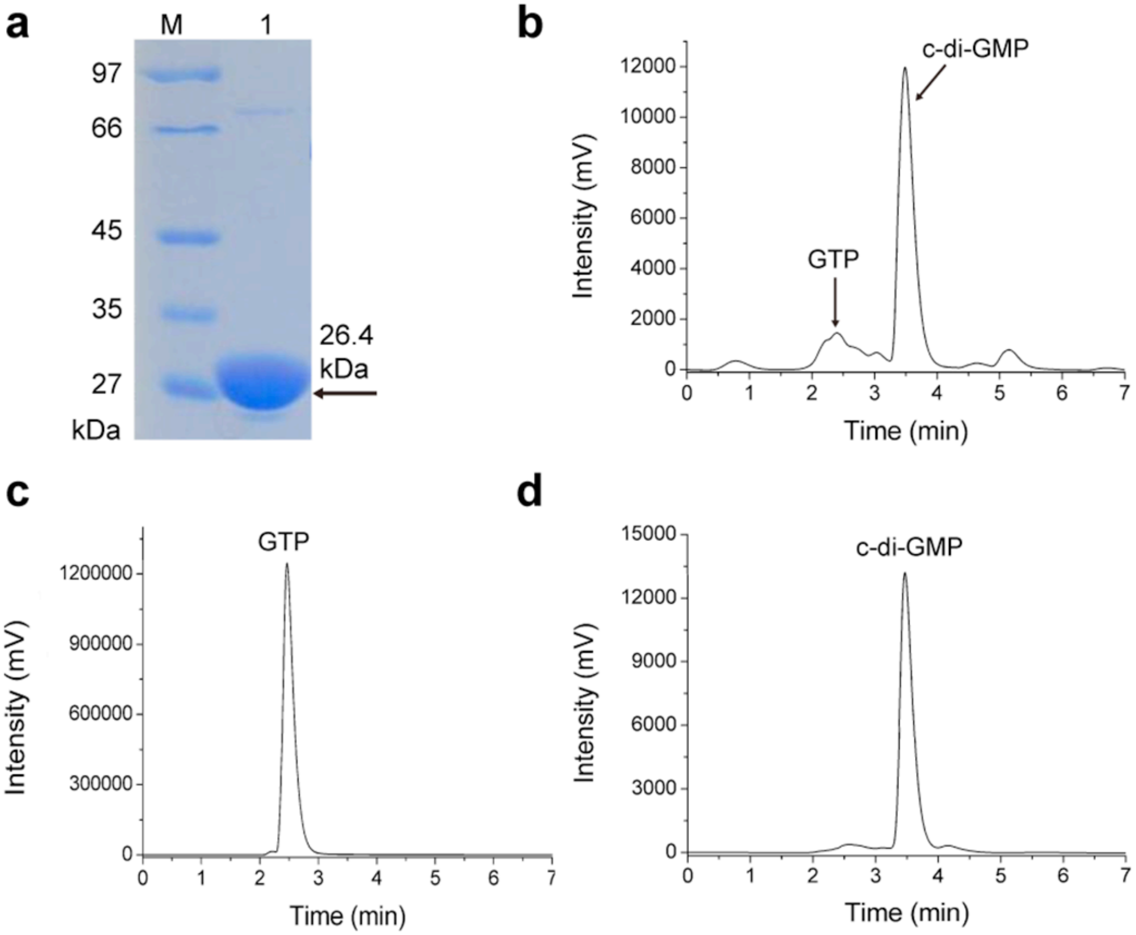

Figure S2. Purified tDgcA is active as a DGC *in vitro*. (**a**) SDS-PAGE analysis of purified tDgcA. Lane M: molecular markers; Lane 1: purified tDgcA. (**b**), (**c**) and (**d**) were the HPLC chromatograms of the *in vitro* reaction products of tDgcA, the GTP standard and c-di-GMP standard, respectively.

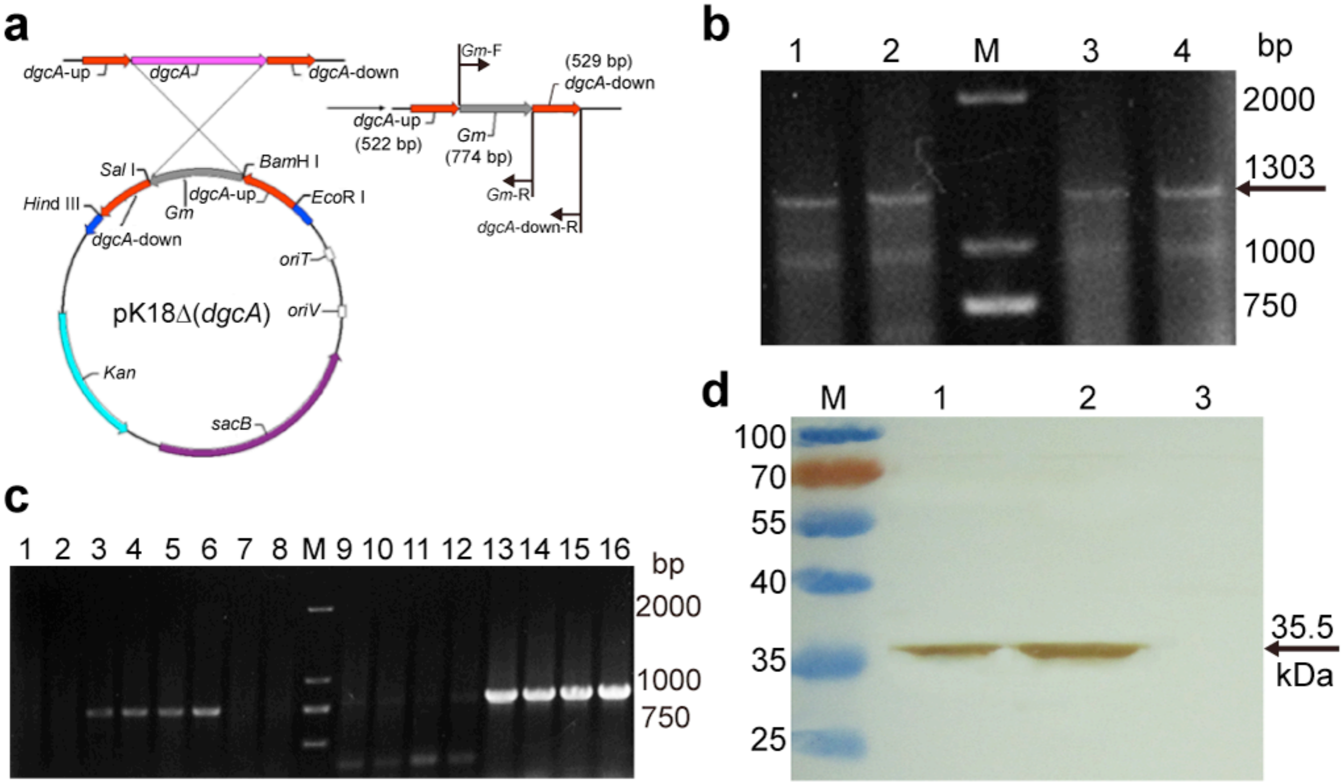

Figure S3. Construction and verification of the WT, Δ*dgcA*, O*dgcA* and C*dgcA* strains. (**a**) Schematic diagram of the gene deletion construct. Allelic homologous recombination strategy was applied with the suicide vector pK18*mobsacB* by replacing *dgcA* with *Gm* gene. (**b**) PCR analyses of the Δ*dgcA* and C*dgcA* strains. Products were amplified using primer pair Gm-F and *dgcA*-down-R from Δ*dgcA* (lanes 1-2) and C*dgcA* strains (lanes 3-4) because both strains contained a replaced *Gm* gene; M: DL2000 DNA ladder. (**c**) PCR confirmation of the WT, Δ*dgcA*, O*dgcA* and C*dgcA* strains. Products were amplified using primer pair Gm-F and Gm-R from WT (lanes 1-2), Δ*dgcA* (lanes 3-4), C*dgcA* (lanes 5-6) and O*dgcA* (lanes 7-8) strains, respectively. Meanwhile, since O*dgcA* and C*dgcA* strains harbored a *kan* gene from pBBR1MCS-*dgcA* plasmid, another primer pair Kan-F and Kan-R was also used to amplify products from WT (lanes 9-10), Δ*dgcA* (lanes 11-12), C*dgcA* (lanes 13-14) and O*dgcA* (lanes 15-16) strains, respectively. M: DL2000 DNA ladder. (**d**) Verification of the DgcA protein in the C*dgcA* and O*dgcA* strains by Western blotting assay with the anti-His mouse monoclonal antibody. M: protein marker; Lane 1: supernatant of C*dgcA*; Line 2: supernatant of O*dgcA*; Line 3: supernatant sample of WT.

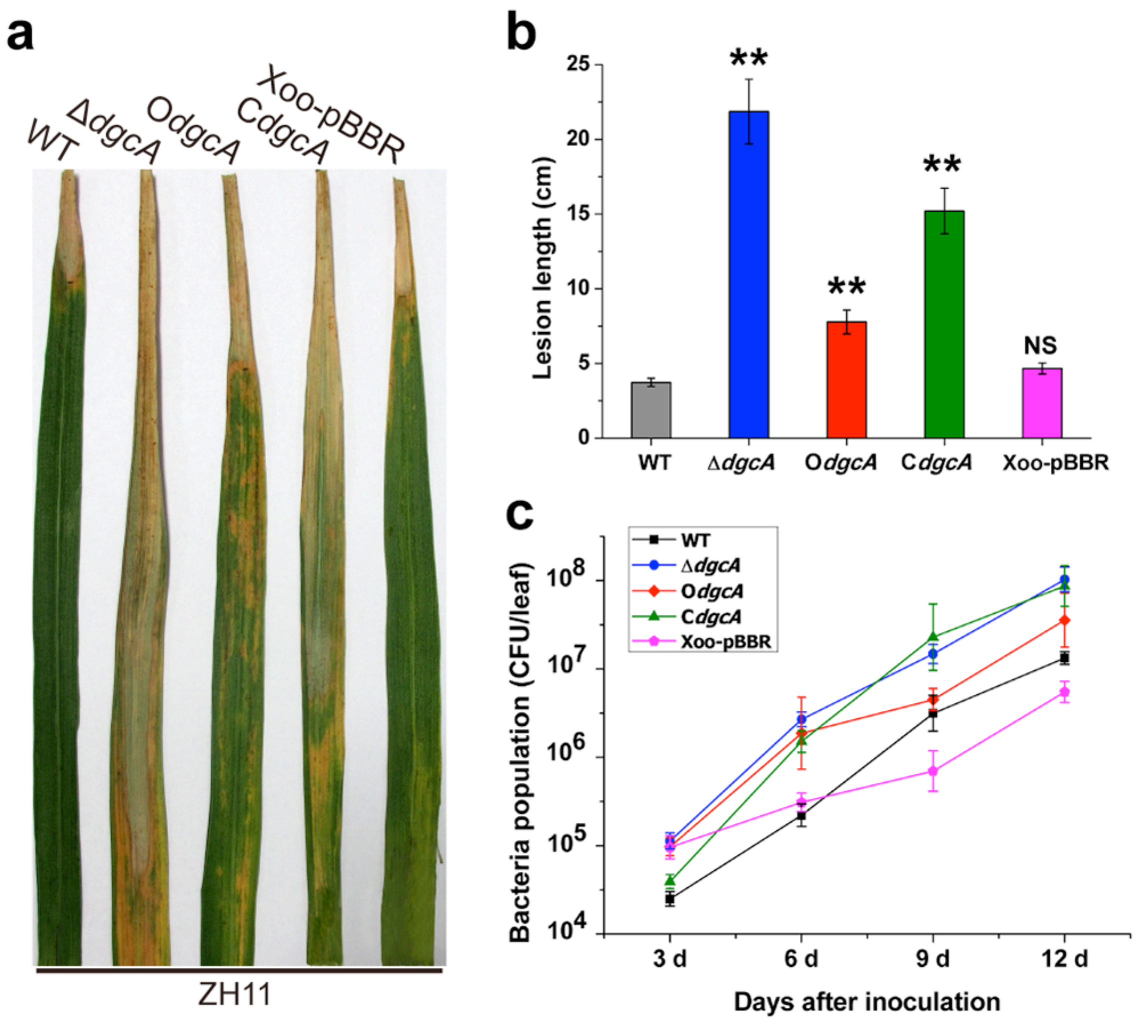

Figure S4. *In Planta* virulence test of the WT, Δ*dgcA*, O*dgcA*, C*dgcA* and Xoo-pBBR strains. (**a**) Leaf segments of rice cultivar Zhonghua 11 (ZH11) showing rice blight symptoms. Typical leaves were photographed after infection for 12 days with the four different strains. (**b**) Measurement of the mean lesion lengths 12 days post inoculation. Leaf without bacterial inoculation were served as a control. (**c**) Determination of bacterial population in rice leaves at four specific time points (3, 6, 9, 12 days after inoculation). The values were means ± standard deviations for triplicate assays. Significances of differences by Student's *t*-test are indicated (**P<0.01; *P<0.05; NS, P>0.05).

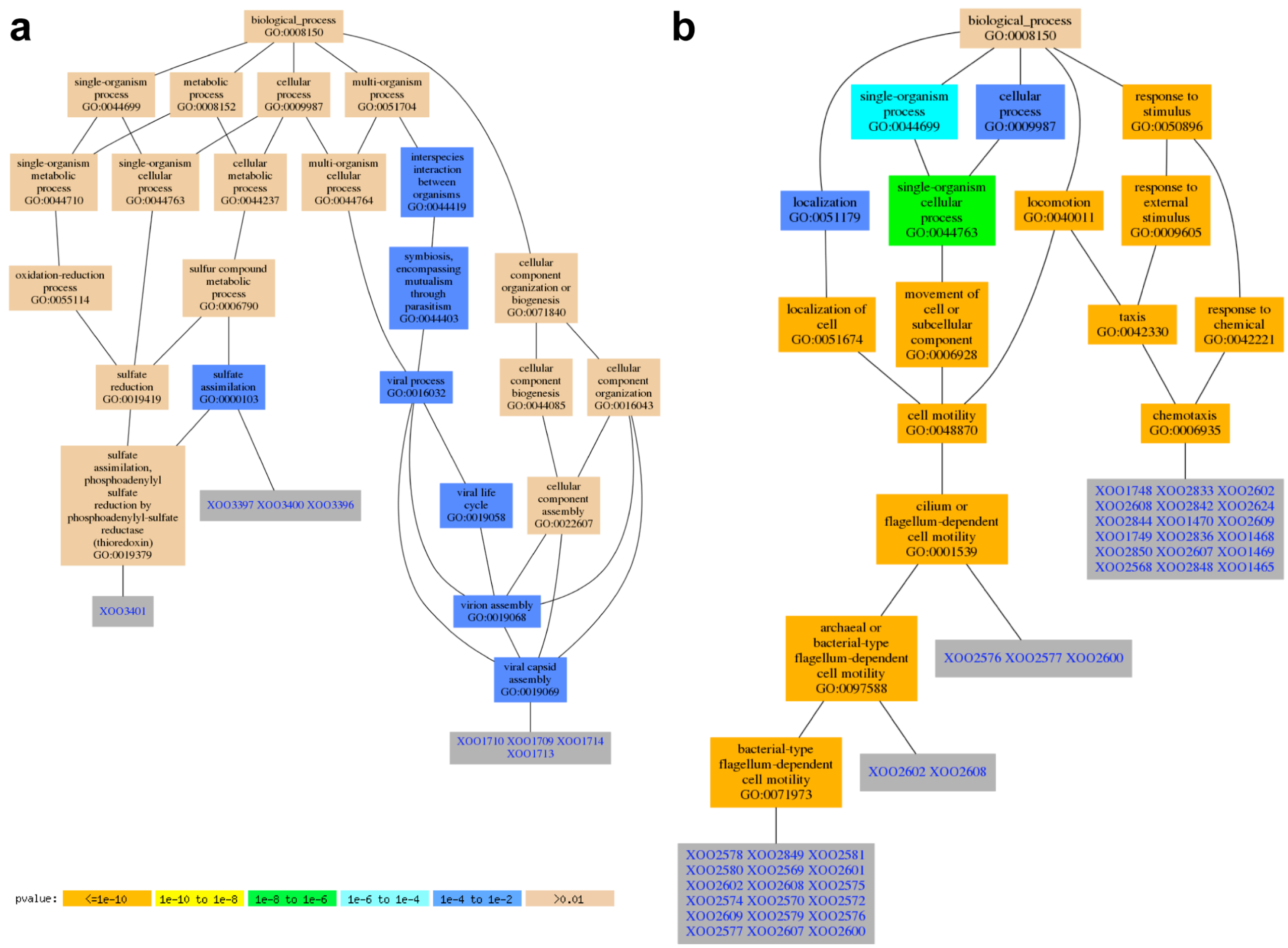

Figure S5. GO tree displays enrich genes associated with certain biological process. (**a**) GO enrichment of up-regulated DGEs. (**b**) GO enrichment of down-regulated DGEs. The color of the nodes indicates their significance according to the p value.

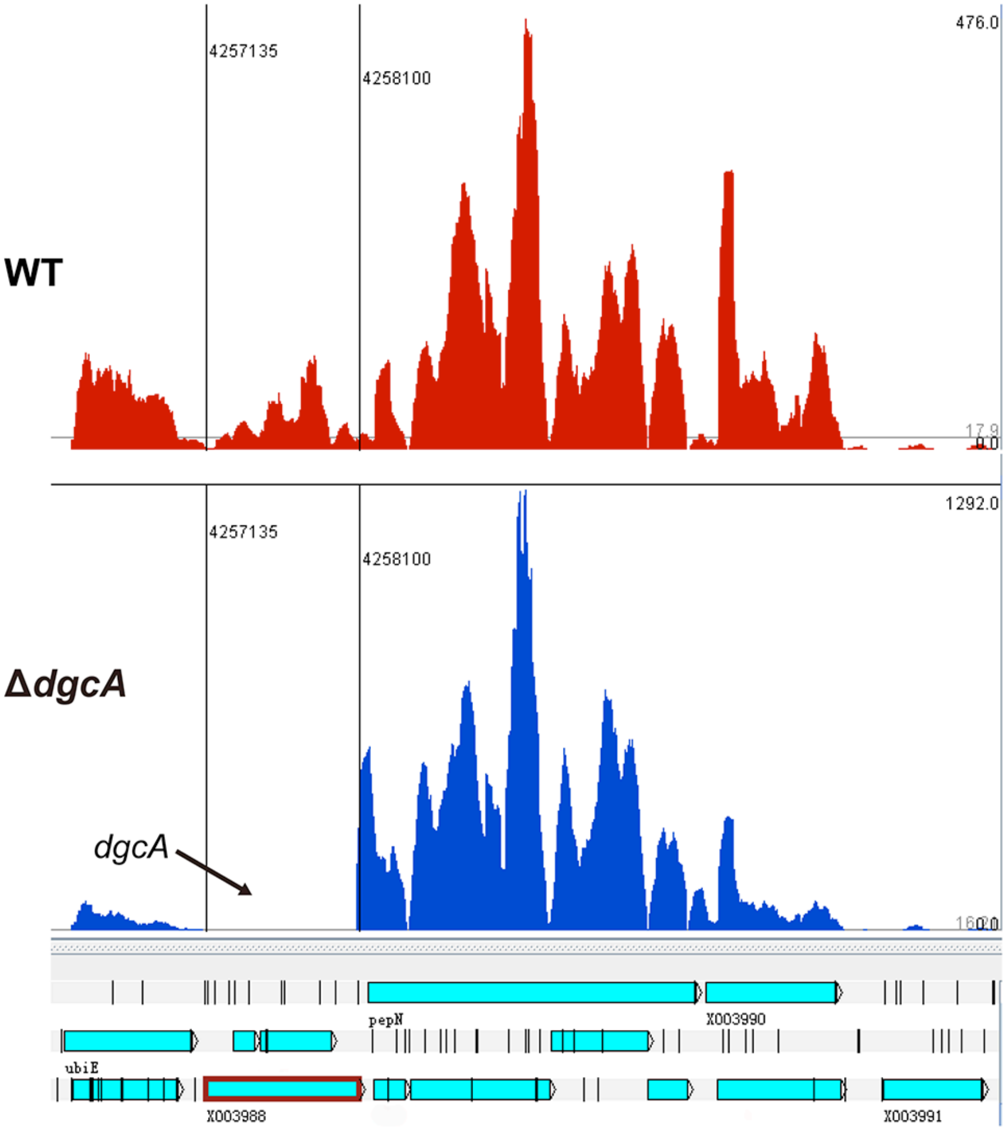

Figure S6. Mapping information of *dgcA* (XOO3988) and its downstream gene *pepN* (XOO3989). The clean reads of WT and Δ*dgcA* were well mapped to the genome by Artemis software.

Section B: supplementary tables

Sample	Raw_reads	Before clean	After clean	Kept_reads
WT	*Xoo*-wt-F.fq	5624657	4917019 + 442496	95.3%
	Xoo-wt-R.fq	5624657	4917019 + 134590	92.1%
Δ*dgcA*	*Xoo*Δ*dgcA*-F.fq	5604713	4827397 + 538387	95.7%
	*Xoo*Δ*dgcA*-R.fq	5604713	4827397 + 99866	90.4%

Table S1. Statistics of reads that cleaned up.

gene	locus_tag	function	length	*ΔdgcA*		WT		*ΔdgcA/WT*		*ΔdgcA/WT*	GeneID	protein_id	product	gi
				reads	RPKM	reads	RPKM	log2 (Fold_ change) normalized	q-value	result				
gi|58579629|ref| YP_198645.1|	XOO0006	hypothetical protein	807	57	20.29	184	64.86	-1.68	7.26E-16	down	3264447	YP_198645.1		58579629
gi|58579652|ref| YP_198668.1|	XOO0029	TonB-dependent receptor	399	181	130.31	390	278.03	-1.09	2.07E-17	down	3263688	YP_198668.1	iroN	58579652
gi|58579655|ref| YP_198671.1|	XOO0032	transposase	1017	827	233.60	88	24.61	3.25	4.29E-149	up	3265582	YP_198671.1		58579655
gi|58579734|ref| YP_198750.1|	XOO0111	hypothetical protein	1629	319	56.25	888	155.06	-1.46	2.87E-60	down	3265706	YP_198750.1		58579734
gi|58579742|ref| YP_198758.1|	XOO0119	methylamine utilization protein	1062	298	80.61	103	27.59	1.55	1.01E-22	up	3265682	YP_198758.1	mauG	58579742
gi|58579745|ref| YP_198761.1|	XOO0122	transposase	1017	885	249.98	81	22.66	3.46	2.6E-168	up	3265699	YP_198761.1		58579745
gi|58579754|ref| YP_198770.1|	XOO0131	VirK protein	516	20	11.13	60	33.08	-1.57	0.0000251	down	3265658	YP_198770.1	virK	58579754
gi|58579759|ref| YP_198775.1|	XOO0136	transposase	696	310	127.95	37	15.12	3.08	6.9E-54	up	3265489	YP_198775.1		58579759
gi|58579784|ref| YP_198800.1|	XOO0161	transposase	1017	811	229.08	99	27.69	3.05	3.74E-138	up	3265509	YP_198800.1		58579784
gi|58579789|ref| YP_198805.1|	XOO0166	ATP-dependent RNA helicase	2598	750	82.93	241	26.39	1.65	2.15E-61	up	3265997	YP_198805.1		58579789
gi|58579790|ref| YP_198806.1|	XOO0167	hypothetical protein	1827	160	25.16	56	8.72	1.53	2.12E-12	up	3266008	YP_198806.1		58579790
gi|58579791|ref| YP_198807.1|	XOO0168	avirulence protein	2157	412	54.87	846	111.56	-1.02	3.93E-33	down	3266007	YP_198807.1	avrBs2	58579791
gi|58579804|ref| YP_198820.1|	XOO0181	IS30 family transposase	114	25	63.00	85	212.09	-1.75	3.05E-08	down	3264145	YP_198820.1		58579804
gi|58579805|ref| YP_198821.1|	XOO0182	transposase	1020	1130	318.25	113	31.51	3.34	3.59E-208	up	3266053	YP_198821.1		58579805
gi|58579823|ref| YP_198839.1|	XOO0200	transposase	438	358	234.80	34	22.08	3.41	8.65E-68	up	3264123	YP_198839.1		58579823
gi|58579824|ref| YP_198840.1|	XOO0201	transposase	279	138	142.09	12	12.23	3.54	1.99E-27	up	3264124	YP_198840.1		58579824
gi|122879005|ref |YP_198921.6|	XOO0282	cellulase	1053	748	204.06	2104	568.36	-1.48	9.95E-144	down	3264040	YP_198921.6	egl	122879005
gi|58579906|ref| YP_198922.1|	XOO0283	cellulase	1131	215	54.61	1582	397.87	-2.87	2.03E-250	down	3264034	YP_198922.1	egl	58579906
gi|58579920|ref| YP_198936.1|	XOO0297	transposase	360	239	190.71	20	15.80	3.59	2.02E-47	up	3264016	YP_198936.1		58579920
gi|58579923|ref| YP_198939.1|	XOO0300	transposase	1053	2029	553.53	423	114.27	2.28	4.19E-251	up	3264013	YP_198939.1		58579923
gi|58579935|ref| YP_198951.1|	XOO0312	acetyltransferase	582	120	59.23	17	8.31	2.83	9.08E-20	up	3263999	YP_198951.1		58579935
gi|58579936|ref| YP_198952.1|	XOO0313	transposase	1017	1090	307.89	83	23.21	3.73	6.5E-219	up	3264000	YP_198952.1		58579936
gi|58579965|ref| YP_198981.1|	XOO0342	transposase	1017	785	221.74	82	22.93	3.27	1.41E-142	up	3263968	YP_198981.1		58579965
gi|58580037|ref| YP_199053.1|	XOO0414	transposase	834	595	204.95	58	19.78	3.37	4.37E-111	up	3263898	YP_199053.1		58580037
gi|58580038|ref| YP_199054.1|	XOO0415	transposase	135	17	36.17	2	4.21	3.10	0.0008133	up	3263897	YP_199054.1		58580038
gi|58580039|ref| YP_199055.1|	XOO0416	membrane-fusion protein	471	180	109.78	43	25.97	2.08	1.05E-20	up	3263896	YP_199055.1		58580039
gi|58580040|ref| YP_199056.1|	XOO0417	catalase	1524	331	62.39	2576	480.80	-2.95	0	down	3263895	YP_199056.1	catB	58580040
gi|58580041|ref| YP_199057.1|	XOO0418	ankyrin-like protein	576	33	16.46	163	80.49	-2.29	6.5E-21	down	3263894	YP_199057.1	ankB	58580041
gi|58580042|ref| YP_199058.1|	XOO0419	hypothetical protein	225	18	22.98	437	552.46	-4.59	3.31E-98	down	3263893	YP_199058.1		58580042
gi|58580168|ref| YP_199184.1|	XOO0545	transposase	135	19	40.43	0	0.00	5.26	0.0000255	up	3263808	YP_199184.1		58580168
gi|58580169|ref| YP_199185.1|	XOO0546	transposase	834	566	194.96	62	21.15	3.20	3.32E-101	up	3263807	YP_199185.1		58580169
gi|58580184|ref| YP_199200.1|	XOO0561	C-type cytochrome biogenesis protein (copper tolerance)	2364	725	88.10	362	43.56	1.02	1.85E-28	up	3263781	YP_199200.1	dsbD	58580184
gi|58580186|ref| YP_199202.1|	XOO0563	transposase	1017	1011	285.57	100	27.97	3.35	4.46E-187	up	3263779	YP_199202.1		58580186
gi|58580234|ref| YP_199250.1|	XOO0611	hypothetical protein	273	217	228.34	16	16.67	3.78	1.12E-44	up	3264294	YP_199250.1		58580234

gi|58580243|ref|YP_199259.1|	XOO0620	transposase	1017	937	264.67	105	29.37	3.17	2.36E-165	up	3264282	YP_199259.1		58580243
gi|58580292|ref|YP_199308.1|	XOO0669	hypothetical protein	1245	619	142.83	198	45.24	1.66	4.2E-51	up	3264150	YP_199308.1		58580292
gi|58580293|ref|YP_199309.1|	XOO0670	DNA polymerase related protein	1107	499	129.49	171	43.94	1.56	6.58E-38	up	3264148	YP_199309.1		58580293
gi|58580310|ref|YP_199326.1|	XOO0687	flagellar motor protein MotB	1134	108	27.36	615	154.26	-2.50	1.46E-84	down	3265958	YP_199326.1	motB	58580310
gi|58580311|ref|YP_199327.1|	XOO0688	flagellar motor protein MotA	855	82	27.55	478	159.02	-2.53	6.63E-67	down	3265959	YP_199327.1	motA	58580311
gi|58580313|ref|YP_199329.1|	XOO0690	methionine sulfoxide reductase B	465	86	53.13	40	24.47	1.12	0.0001054	up	3265962	YP_199329.1		58580313
gi|58580339|ref|YP_199355.1|	XOO0716	hypothetical protein	558	134	68.99	65	33.13	1.06	0.0000026	up	3261775	YP_199355.1		58580339
gi|58580360|ref|YP_199376.1|	XOO0737	histidine kinase-response regulator hybrid protein	150	37	70.86	10	18.96	1.90	0.0001657	up	3265902	YP_199376.1	styS	58580360
gi|58580362|ref|YP_199378.1|	XOO0739	transposase	1017	780	220.32	87	24.33	3.18	4.3E-138	up	3265906	YP_199378.1		58580362
gi|58580370|ref|YP_199386.1|	XOO0747	hypothetical protein	474	221	133.94	868	520.89	-1.96	1.41E-88	down	3265924	YP_199386.1		58580370
gi|58580371|ref|YP_199387.1|	XOO0748	hypothetical protein	576	832	414.94	4951	2444.97	-2.56	0	down	3266193	YP_199387.1		58580371
gi|58580409|ref|YP_199425.1|	XOO0786	IS1478 transposase	141	38	77.42	81	163.41	-1.08	0.0003043	down	3266182	YP_199425.1		58580409
gi|58580416|ref|YP_199432.1|	XOO0793	glucose-1-phosphate thymidylyltransferase	984	416	121.45	158	45.67	1.41	1.76E-27	up	3265850	YP_199432.1	rmlA	58580416
gi|58580417|ref|YP_199433.1|	XOO0794	dTDP-4-dehydrorhamnose 3,5-epimerase	558	580	298.59	246	125.40	1.25	1E-31	up	3265851	YP_199433.1	rmlC	58580417
gi|58580418|ref|YP_199434.1|	XOO0795	dTDP-4-dehydrorhamnose reductase	909	374	118.19	138	43.18	1.45	7.25E-26	up	3265853	YP_199434.1	rmlD	58580418
gi|122879050|ref|YP_199451.6|	XOO0812	ribosomal-protein-alanine acetyltransferase	474	44	26.67	89	53.41	-1.00	0.0003678	down	3264625	YP_199451.6	rimI	122879050
gi|58580476|ref|YP_199492.1|	XOO0853	general secretion pathway protein K	906	73	23.15	215	67.50	-1.54	1.96E-16	down	3264580	YP_199492.1	xpsK	58580476
gi|58580497|ref|YP_199513.1|	XOO0874	transposase	627	659	301.93	72	32.66	3.21	8.5E-118	up	3264559	YP_199513.1		58580497
gi|58580498|ref|YP_199514.1|	XOO0875	transposase	282	25	25.47	3	3.03	3.07	0.0000389	up	3264558	YP_199514.1		58580498
gi|58580502|ref|YP_199518.1|	XOO0879	hypothetical protein	441	31	20.19	79	50.96	-1.34	0.0000181	down	3264554	YP_199518.1		58580502
gi|58580540|ref|YP_199556.1|	XOO0917	IS1595 transposase	783	600	220.13	71	25.79	3.09	7.3E-104	up	3264461	YP_199556.1		58580540
gi|58580555|ref|YP_199571.1|	XOO0932	transposase	693	540	223.85	62	25.45	3.14	9.06E-95	up	3264428	YP_199571.1		58580555
gi|58580579|ref|YP_199595.1|	XOO0956	transposase	1017	1067	301.39	92	25.73	3.55	1.12E-206	up	3264778	YP_199595.1		58580579
gi|58580580|ref|YP_199596.1|	XOO0957	H+ translocating pyrophosphate synthase	339	566	479.63	1394	1169.68	-1.29	1.66E-77	down	3265203	YP_199596.1		58580580
gi|58580583|ref|YP_199599.1|	XOO0960	transposase	834	791	272.46	70	23.87	3.51	3.36E-152	up	3265435	YP_199599.1		58580583
gi|58580602|ref|YP_199618.1|	XOO0979	disulfide bond formation protein B	525	111	60.74	241	130.58	-1.10	2.91E-11	down	3264748	YP_199618.1	dsbB	58580602
gi|58580603|ref|YP_199619.1|	XOO0980	IS1478 transposase	489	72	42.30	35	20.36	1.05	0.0008319	up	3264746	YP_199619.1		58580603
gi|58580612|ref|YP_199628.1|	XOO0989	transposase	966	479	142.44	51	15.02	3.25	9.69E-87	up	3264735	YP_199628.1		58580612
gi|58580626|ref|YP_199642.1|	XOO1003	transposase	954	773	232.77	88	26.24	3.15	1.01E-135	up	3264710	YP_199642.1		58580626
gi|58580646|ref|YP_199662.1|	XOO1023	transposase	1017	1016	286.99	104	29.09	3.30	1.17E-185	up	3264667	YP_199662.1		58580646
gi|58580676|ref|YP_199692.1|	XOO1053	transposase	951	716	216.28	59	17.65	3.62	6.31E-141	up	3264877	YP_199692.1		58580676
gi|58580685|ref|YP_199701.1|	XOO1062	molybdopterin-converting factor subunit 1	246	28	32.70	67	77.47	-1.24	0.0002218	down	3264868	YP_199701.1	moaD	58580685

gi|58580712|ref|YP_199728.1|	XOO1089	ISXo8 transposase	288	27	26.93	79	78.03	-1.53	1.54E-06	down	3264965	YP_199728.1		58580712
gi|58580781|ref|YP_199797.1|	XOO1158	fimbrial assembly membrane protein	759	1954	739.55	4192	1571.02	-1.09	2.96E-177	down	3265148	YP_199797.1	pilN	58580781
gi|58580782|ref|YP_199798.1|	XOO1159	fimbrial assembly membrane protein	666	1032	445.14	2173	928.09	-1.06	1.17E-88	down	3265149	YP_199798.1	pilO	58580782
gi|58580783|ref|YP_199799.1|	XOO1160	fimbrial assembly protein	534	1112	598.21	2309	1229.94	-1.04	2.84E-91	down	3265147	YP_199799.1	pilP	58580783
gi|58580784|ref|YP_199800.1|	XOO1161	fimbrial assembly protein	2022	5342	758.95	13299	1870.86	-1.30	0	down	3265146	YP_199800.1	pilQ	58580784
gi|58580829|ref|YP_199845.1|	XOO1206	transposase	1062	1199	324.33	304	81.42	1.99	4.8E-126	up	3265062	YP_199845.1		58580829
gi|58580840|ref|YP_199856.1|	XOO1217	hypothetical protein	360	1	0.80	15	11.85	-3.89	0.0005981	down	3263546	YP_199856.1		58580840
gi|122879081|ref|YP_199912.6|	XOO1273	quinol oxidase subunit I	1401	1099	225.34	2291	465.15	-1.05	2.28E-91	down	3263610	YP_199912.6		122879081
gi|58580897|ref|YP_199913.1|	XOO1274	quinol oxidase subunit II	1020	836	235.45	1764	491.93	-1.06	1.88E-72	down	3263609	YP_199913.1	qxtB	58580897
gi|58580938|ref|YP_199954.1|	XOO1315	transposase	954	853	256.86	69	20.57	3.64	1.34E-168	up	3265486	YP_199954.1		58580938
gi|58580943|ref|YP_199959.1|	XOO1320	transposase	1017	806	227.67	63	17.62	3.69	6.23E-161	up	3265879	YP_199959.1		58580943
gi|58580979|ref|YP_199995.1|	XOO1356	transporter	1197	33	7.92	8	1.90	2.06	0.0001798	up	3263801	YP_199995.1	yceE	58580979
gi|122879096|ref|YP_200019.6|	XOO1380	HrpX protein	1431	399	80.10	1117	222.03	-1.47	3.34E-76	down	3266082	YP_200019.6	hrpXct	122879096
gi|58581015|ref|YP_200031.1|	XOO1392	IS1404 transposase	177	34	55.18	3	4.82	3.52	2.25E-07	up	3266164	YP_200031.1		58581015
gi|58581016|ref|YP_200032.1|	XOO1393	transposase	1017	695	196.31	63	17.62	3.48	7.83E-133	up	3266138	YP_200032.1		58581016
gi|58581022|ref|YP_200038.1|	XOO1399	IS1479 transposase	216	72	95.76	162	213.34	-1.16	2.16E-08	down	3266130	YP_200038.1		58581022
gi|58581036|ref|YP_200052.1|	XOO1413	peptidase	732	53	20.80	21	8.16	1.35	0.0004659	up	3266173	YP_200052.1		58581036
gi|58581067|ref|YP_200083.1|	XOO1444	transposase	999	683	196.40	60	17.08	3.52	6.97E-132	up	3262169	YP_200083.1		58581067
gi|122879106|ref|YP_200098.6|	XOO1459	endonuclease V	714	147	59.14	62	24.70	1.26	9.06E-09	up	3262184	YP_200098.6	nfi	122879106
gi|161899005|ref|YP_200104.2|	XOO1465	chemotaxis-specific methylesterase	1122	105	26.88	695	176.20	-2.71	1.64E-104	down	3262190	YP_200104.2	cheB	161899005
gi|58581089|ref|YP_200105.1|	XOO1466	response regulator for chemotaxis	828	45	15.61	451	154.93	-3.31	2.61E-82	down	3262191	YP_200105.1	cheR	58581089
gi|58581090|ref|YP_200106.1|	XOO1467	response regulator	1302	46	10.15	720	157.30	-3.95	3.46E-149	down	3262192	YP_200106.1	vieA	58581090
gi|58581091|ref|YP_200107.1|	XOO1468	chemotaxis protein	516	125	69.59	2540	1400.19	-4.33	0	down	3262193	YP_200107.1	cheW	58581091
gi|58581092|ref|YP_200108.1|	XOO1469	chemotaxis protein	2169	362	47.94	6596	865.01	-4.17	0	down	3262194	YP_200108.1	mcp	58581092
gi|58581093|ref|YP_200109.1|	XOO1470	chemotaxis histidine protein kinase	2022	130	18.47	1872	263.35	-3.83	0	down	3262195	YP_200109.1	cheA	58581093
gi|58581094|ref|YP_200110.1|	XOO1471	hypothetical protein	330	23	20.02	282	243.07	-3.60	2.34E-55	down	3262196	YP_200110.1		58581094
gi|58581104|ref|YP_200120.1|	XOO1481	hypothetical protein	573	62	31.08	23	11.42	1.44	0.0000554	up	3262206	YP_200120.1	creD	58581104
gi|122879107|ref|YP_200126.6|	XOO1487	cysteine protease	813	381	134.62	1487	520.26	-1.95	3.95E-150	down	3262211	YP_200126.6		122879107
gi|58581138|ref|YP_200154.1|	XOO1515	hypothetical protein	216	77	102.41	34	44.77	1.19	0.0001117	up	3262240	YP_200154.1		58581138
gi|58581174|ref|YP_200190.1|	XOO1551	hypothetical protein	2523	110	12.52	394	44.42	-1.83	4.38E-37	down	3262588	YP_200190.1		58581174
gi|58581209|ref|YP_200225.1|	XOO1586	pilin	507	2538	1438.04	5781	3243.38	-1.17	4.71E-276	down	3263396	YP_200225.1	pilE	58581209
gi|58581303|ref|YP_200319.1|	XOO1680	phage-related integrase	1185	2761	669.32	89	21.36	4.97	0	up	3262398	YP_200319.1	int	58581303
gi|58581304|ref|YP_200320.1|	XOO1681	hypothetical protein	261	1351	1486.97	10	10.90	7.09	1.31E-305	up	3262399	YP_200320.1		58581304
gi|58581305|ref|YP_200321.1|	XOO1682	hypothetical protein	276	2264	2356.43	26	26.80	6.46	0	up	3262400	YP_200321.1		58581305
gi|58581306|ref|YP_200322.1|	XOO1683	hypothetical protein	411	4443	3105.43	42	29.07	6.74	0	up	3262401	YP_200322.1		58581306
gi|58581307|ref|YP_200323.1|	XOO1684	hypothetical protein	279	435	447.89	12	12.23	5.19	1.62E-102	up	3262350	YP_200323.1		58581307

gi|58581308|ref|YP_200324.1|	XOO1685	hypothetical protein	2697	7494	798.22	93	9.81	6.35	0	up	3262351	YP_200324.1		58581308
gi|58581309|ref|YP_200325.1|	XOO1686	hypothetical protein	213	2318	3126.23	20	26.71	6.87	0	up	3262352	YP_200325.1		58581309
gi|58581310|ref|YP_200326.1|	XOO1687	hypothetical protein	327	3530	3101.09	35	30.45	6.67	0	up	3262373	YP_200326.1		58581310
gi|58581311|ref|YP_200327.1|	XOO1688	hypothetical protein	438	546	358.10	239	155.21	1.21	2.84E-28	up	3262282	YP_200327.1		58581311
gi|58581312|ref|YP_200328.1|	XOO1689	phage-related tail protein	987	3912	1138.60	68	19.60	5.86	0	up	3262283	YP_200328.1		58581312
gi|58581313|ref|YP_200329.1|	XOO1690	phage-related tail protein	402	823	588.11	5	3.54	7.38	8.03E-183	up	3262284	YP_200329.1		58581313
gi|58581314|ref|YP_200330.1|	XOO1691	phage-related tail protein	2871	8685	869.01	77	7.63	6.83	0	up	3261530	YP_200330.1		58581314
gi|58581315|ref|YP_200331.1|	XOO1692	hypothetical protein	114	831	2094.03	4	9.98	7.71	1.25E-179	up	3261741	YP_200331.1		58581315
gi|58581316|ref|YP_200332.1|	XOO1693	phage-related tail protein	303	2498	2368.30	10	9.39	7.98	0	up	3261614	YP_200332.1		58581316
gi|58581317|ref|YP_200333.1|	XOO1694	phage-related tail protein	510	20017	11275.00	120	66.93	7.40	0	up	3261854	YP_200333.1		58581317
gi|58581318|ref|YP_200334.1|	XOO1695	phage-related tail protein	1167	41657	10254.27	166	40.46	7.99	0	up	3261852	YP_200334.1		58581318
gi|58581319|ref|YP_200335.1|	XOO1696	phage-related baseplate protein	360	2534	2022.05	4	3.16	9.32	0	up	3261746	YP_200335.1		58581319
gi|58581320|ref|YP_200336.1|	XOO1697	phage-related baseplate protein	564	5645	2875.23	10	5.04	9.16	0	up	3261759	YP_200336.1		58581320
gi|58581321|ref|YP_200337.1|	XOO1698	hypothetical protein	1506	8209	1565.86	90	17.00	6.53	0	up	3261761	YP_200337.1		58581321
gi|58581322|ref|YP_200338.1|	XOO1699	phage-related tail protein	570	4036	2034.06	26	12.97	7.29	0	up	3263345	YP_200338.1		58581322
gi|58581323|ref|YP_200339.1|	XOO1700	phage-related baseplate assembly protein	954	2999	903.06	17	5.07	7.48	0	up	3263341	YP_200339.1		58581323
gi|58581324|ref|YP_200340.1|	XOO1701	hypothetical protein	843	9240	3148.71	2153	726.47	2.12	0	up	3263139	YP_200340.1		58581324
gi|58581325|ref|YP_200341.1|	XOO1702	phage-related tail protein	447	1316	845.74	18	11.45	6.21	0	up	3263140	YP_200341.1		58581325
gi|58581326|ref|YP_200342.1|	XOO1703	phage-related tail protein	453	3714	2355.22	32	20.09	6.87	0	up	3263143	YP_200342.1		58581326
gi|58581327|ref|YP_200343.1|	XOO1704	hypothetical protein	489	5392	3167.59	36	20.94	7.24	0	up	3263144	YP_200343.1		58581327
gi|58581328|ref|YP_200344.1|	XOO1705	phage-related lytic enzyme	642	8587	3842.32	36	15.95	7.91	0	up	3263145	YP_200344.1	lys	58581328
gi|58581329|ref|YP_200345.1|	XOO1706	hypothetical protein	291	3083	3043.46	20	19.55	7.28	0	up	3263156	YP_200345.1		58581329
gi|58581330|ref|YP_200346.1|	XOO1707	hypothetical protein	357	2030	1633.49	24	19.12	6.42	0	up	3263157	YP_200346.1		58581330
gi|58581331|ref|YP_200347.1|	XOO1708	phage-related tail protein	210	671	917.89	3	4.06	7.82	7.26E-144	up	3263162	YP_200347.1		58581331
gi|58581332|ref|YP_200348.1|	XOO1709	phage-related capsid completion protein	378	3129	2377.94	19	14.30	7.38	0	up	3263164	YP_200348.1		58581332
gi|58581333|ref|YP_200349.1|	XOO1710	phage-related terminase	720	24742	9871.65	105	41.48	7.89	0	up	3263277	YP_200349.1		58581333
gi|58581335|ref|YP_200351.1|	XOO1712	phage-related major capsid protein	1017	38578	10896.99	169	47.27	7.85	0	up	3263280	YP_200351.1		58581335
gi|58581336|ref|YP_200352.1|	XOO1713	phage-related capsid scaffold protein	843	41502	14142.59	130	43.87	8.33	0	up	3263089	YP_200352.1		58581336
gi|58581337|ref|YP_200353.1|	XOO1714	phage-related terminase	1785	5593	900.11	23	3.67	7.94	0	up	3263090	YP_200353.1		58581337
gi|58581338|ref|YP_200354.1|	XOO1715	phage-related capsid packaging protein	1026	3190	893.16	19	5.27	7.41	0	up	3263093	YP_200354.1		58581338
gi|122879126|ref|YP_200356.6|	XOO1717	site-specific DNA-methyltransferase	702	5670	2320.24	52	21.07	6.78	0	up	3263095	YP_200356.6		122879126
gi|58581341|ref|YP_200357.1|	XOO1718	hypothetical protein	558	851	438.11	282	143.75	1.61	6.63E-67	up	3263096	YP_200357.1		58581341
gi|58581349|ref|YP_200365.1|	XOO1726	transposase	1143	2524	634.35	829	206.31	1.62	6.34E-199	up	3263104	YP_200365.1		58581349
gi|58581365|ref|YP_200381.1|	XOO1742	hypothetical protein	813	151	53.35	65	22.74	1.23	1.09E-08	up	3263121	YP_200381.1		58581365

gi|122879129|ref |YP_200385.6|	XOO1746	pilus protein	402	2885	2061.61	8416	5955.00	-1.53	0	down	3263065	YP_200385.6	pilG	122879129
gi|58581370|ref| YP_200386.1|	XOO1747	PilH protein	363	178	140.86	586	459.19	-1.70	7.97E-50	down	3263066	YP_200386.1	pilH	58581370
gi|58581371|ref| YP_200387.1|	XOO1748	pilus biogenesis protein	534	531	285.65	1486	791.55	-1.47	4.66E-101	down	3263067	YP_200387.1	pilI	58581371
gi|58581372|ref| YP_200388.1|	XOO1749	pilus biogenesis protein	2037	2360	332.82	4770	666.09	-1.00	3.01E-176	down	3263068	YP_200388.1	pilJ	58581372
gi|58581377|ref| YP_200393.1|	XOO1754	ribosomal protein alanine acetyltransferase	624	106	48.80	221	100.74	-1.05	1.37E-09	down	3263059	YP_200393.1	rimJ	58581377
gi|58581407|ref| YP_200423.1|	XOO1784	TonB-dependent receptor	2712	9484	1004.59	20033	2101.16	-1.06	0	down	3262953	YP_200423.1	iroN	58581407
gi|58581487|ref| YP_200503.1|	XOO1864	polyketide synthase	759	95	35.96	47	17.61	1.03	0.0001431	up	3262722	YP_200503.1	frnE	58581487
gi|58581510|ref| YP_200526.1|	XOO1887	hypothetical protein	267	28	30.13	67	71.38	-1.24	0.0002218	down	3262659	YP_200526.1		58581510
gi|58581572|ref| YP_200588.1|	XOO1949	transposase	462	189	117.52	15	9.24	3.67	2.94E-38	up	3265290	YP_200588.1		58581572
gi|58581601|ref| YP_200617.1|	XOO1978	pili assembly chaperone	732	15	5.89	152	59.07	-3.33	2E-28	down	3263449	YP_200617.1		58581601
gi|58581604|ref| YP_200620.1|	XOO1981	pili assembly chaperone	783	632	231.87	1355	492.24	-1.09	8.23E-58	down	3265302	YP_200620.1	ecpD	58581604
gi|58581605|ref| YP_200621.1|	XOO1982	protein U	585	478	234.73	1077	523.68	-1.16	4.97E-51	down	3265361	YP_200621.1	pru	58581605
gi|58581621|ref| YP_200637.1|	XOO1998	MarR family transcriptional regulator	480	200	119.70	98	58.07	1.04	8.13E-09	up	3263423	YP_200637.1	yybA	58581621
gi|58581633|ref| YP_200649.1|	XOO2010	transposase	135	20	42.56	1	2.11	4.34	0.0000296	up	3265746	YP_200649.1		58581633
gi|58581634|ref| YP_200650.1|	XOO2011	transposase	816	645	227.07	67	23.36	3.28	1.38E-117	up	3262142	YP_200650.1		58581634
gi|58581710|ref| YP_200726.1|	XOO2087	transposase	462	168	104.46	15	9.24	3.50	6.39E-33	up	3262092	YP_200726.1		58581710
gi|58581711|ref| YP_200727.1|	XOO2088	transposase	579	655	324.98	61	29.97	3.44	3.95E-124	up	3262091	YP_200727.1		58581711
gi|58581723|ref| YP_200739.1|	XOO2100	transposase	1017	603	170.33	64	17.90	3.25	4.32E-109	up	3262080	YP_200739.1		58581723
gi|58581725|ref| YP_200741.1|	XOO2102	ISXo8 transposase	297	34	32.89	77	73.75	-1.17	0.0001678	down	3262078	YP_200741.1		58581725
gi|58581730|ref| YP_200746.1|	XOO2107	transposase	1017	625	176.54	67	18.74	3.24	1.32E-112	up	3262074	YP_200746.1		58581730
gi|58581781|ref| YP_200797.1|	XOO2158	transposase	1017	884	249.70	92	25.73	3.28	1.18E-160	up	3266038	YP_200797.1		58581781
gi|58581826|ref| YP_200842.1|	XOO2203	hypothetical protein	606	229	108.56	1901	892.30	-3.04	0	down	3265446	YP_200842.1		58581826
gi|58581827|ref| YP_200843.1|	XOO2204	methyl-accepting chemotaxis protein	1893	906	137.49	12583	1890.76	-3.78	0	down	3265436	YP_200843.1	tlpC	58581827
gi|58581850|ref| YP_200866.1|	XOO2227	two-component system regulatory protein	567	310	157.06	627	314.55	-1.00	6.06E-24	down	3265481	YP_200866.1	regR	58581850
gi|58581858|ref| YP_200874.1|	XOO2235	hypothetical protein	762	41	15.46	14	5.23	1.56	0.0006001	up	3266086	YP_200874.1		58581858
gi|58581908|ref| YP_200924.1|	XOO2285	transposase	1017	679	191.79	62	17.34	3.47	1.71E-129	up	3265936	YP_200924.1		58581908
gi|58581924|ref| YP_200940.1|	XOO2301	transposase	1017	637	179.93	50	13.98	3.69	2.84E-127	up	3261700	YP_200940.1		58581924
gi|58581925|ref| YP_200941.1|	XOO2302	hypothetical protein	306	151	141.76	43	39.97	1.83	7.02E-15	up	3261536	YP_200941.1		58581925
gi|58581971|ref| YP_200987.1|	XOO2348	hypothetical protein	189	33	50.16	367	552.34	-3.46	1.32E-69	down	3261810	YP_200987.1		58581971
gi|58581977|ref| YP_200993.1|	XOO2354	hypothetical protein	1857	191	29.55	1462	223.94	-2.92	4.6E-236	down	3261850	YP_200993.1		58581977
gi|58582000|ref| YP_201016.1|	XOO2377	cation efflux system protein	4575	121	7.60	61	3.79	1.00	0.0000227	up	3261773	YP_201016.1	czcA	58582000
gi|58582102|ref| YP_201118.1|	XOO2479	site-specific recombinase	375	15	11.49	1	0.76	3.92	0.0005395	up	3261716	YP_201118.1		58582102
gi|58582139|ref| YP_201155.1|	XOO2516	transposase	1017	1028	290.38	96	26.85	3.43	4.57E-194	up	3261572	YP_201155.1		58582139
gi|58582181|ref| YP_201197.1|	XOO2558	chemotaxis protein	1278	160	35.96	3031	674.62	-4.23	0	down	3261621	YP_201197.1	mcp	58582181
gi|58582182|ref| YP_201198.1|	XOO2559	hypothetical protein	288	8	7.98	103	101.73	-3.67	4.85E-21	down	3261622	YP_201198.1		58582182

gi|122879199|ref |YP_201200.6|	XOO2561	hypothetical protein	2517	71	8.10	597	67.47	-3.06	2.99E-101	down	3261624	YP_201200.6		122879199
gi|58582188|ref| YP_201204.1|	XOO2565	hypothetical protein	333	112	96.62	1448	1236.88	-3.68	6.83E-285	down	3261628	YP_201204.1		58582188
gi|58582189|ref| YP_201205.1|	XOO2566	flagellar protein	312	166	152.84	2923	2664.87	-4.12	0	down	3261629	YP_201205.1	flgM	58582189
gi|58582190|ref| YP_201206.1|	XOO2567	flagellar basal body P-ring biosynthesis protein FlgA	645	105	46.76	881	388.52	-3.05	8.41E-149	down	3261630	YP_201206.1	flgA	58582190
gi|58582191|ref| YP_201207.1|	XOO2568	chemotaxis protein	945	26	7.90	703	211.60	-4.74	2.22E-159	down	3261631	YP_201207.1	cheV	58582191
gi|58582192|ref| YP_201208.1|	XOO2569	flagellar basal-body rod protein FlgB	399	124	89.28	676	481.92	-2.43	3.25E-90	down	3261632	YP_201208.1	flgB	58582192
gi|58582193|ref| YP_201209.1|	XOO2570	flagellar basal body rod protein FlgC	384	213	159.34	916	678.53	-2.09	1.6E-101	down	3261633	YP_201209.1	flgC	58582193
gi|58582194|ref| YP_201210.1|	XOO2571	flagellar basal body rod modification protein	666	152	65.56	1300	555.23	-3.08	5.6E-221	down	3261634	YP_201210.1	flgD	58582194
gi|58582195|ref| YP_201211.1|	XOO2572	flagellar hook protein FlgE	1224	375	88.01	3719	864.27	-3.30	0	down	3261635	YP_201211.1	flgE	58582195
gi|161898986|ref |YP_201213.2|	XOO2574	flagellar basal body rod protein FlgF	756	137	52.06	924	347.66	-2.74	4.4E-140	down	3261637	YP_201213.2	flgF	161898986
gi|161898985|ref |YP_201214.2|	XOO2575	flagellar basal body rod protein FlgG	786	344	125.73	1946	704.24	-2.49	1.97E-264	down	3261638	YP_201214.2	flgG	161898985
gi|58582199|ref| YP_201215.1|	XOO2576	flagellar basal body L-ring protein	693	212	87.88	1503	616.92	-2.81	1.86E-233	down	3261639	YP_201215.1	flgH	58582199
gi|58582200|ref| YP_201216.1|	XOO2577	flagellar basal body P-ring biosynthesis protein FlgA	1119	152	39.02	1061	269.70	-2.79	1.06E-163	down	3261640	YP_201216.1	flgI	58582200
gi|58582201|ref| YP_201217.1|	XOO2578	flagellar rod assembly protein/muramidase FlgJ	1179	134	32.65	799	192.77	-2.56	7.55E-113	down	3261641	YP_201217.1	flgJ	58582201
gi|58582202|ref| YP_201218.1|	XOO2579	flagellar hook-associated protein FlgK	1875	193	29.57	938	142.30	-2.27	7.02E-115	down	3261642	YP_201218.1	flgK	58582202
gi|58582203|ref| YP_201219.1|	XOO2580	flagellar hook-associated protein FlgL	1206	122	29.06	807	190.34	-2.71	3.69E-121	down	3261643	YP_201219.1	flgL	58582203
gi|161898984|ref |YP_201220.2|	XOO2581	flagellin	1200	408	97.67	8854	2098.75	-4.43	0	down	3261644	YP_201220.2	fliC	161898984
gi|58582205|ref| YP_201221.1|	XOO2582	flagellar protein	1326	411	89.04	4465	957.81	-3.43	0	down	3261645	YP_201221.1	fliD	58582205
gi|58582206|ref| YP_201222.1|	XOO2583	flagellar protein	414	84	58.29	681	467.90	-3.00	1.72E-113	down	3261646	YP_201222.1	fliS	58582206
gi|58582207|ref| YP_201223.1|	XOO2584	hypothetical protein	306	108	101.39	812	754.81	-2.90	2.14E-130	down	3261647	YP_201223.1		58582207
gi|58582208|ref| YP_201224.1|	XOO2585	hypothetical protein	567	35	17.73	212	106.35	-2.58	7.24E-31	down	3261868	YP_201224.1		58582208
gi|58582210|ref| YP_201226.1|	XOO2587	RNA polymerase sigma-54 factor	1404	1526	312.23	4140	838.76	-1.43	2.58E-267	down	3261870	YP_201226.1	rpoN	58582210
gi|58582211|ref| YP_201227.1|	XOO2588	response regulator	381	552	416.20	1129	842.89	-1.02	1.5E-43	down	3261871	YP_201227.1		58582211
gi|58582214|ref| YP_201230.1|	XOO2591	acyl carrier protein	324	175	155.16	377	330.98	-1.09	7.42E-17	down	3261874	YP_201230.1	acp	58582214
gi|58582215|ref| YP_201231.1|	XOO2592	3-oxoacyl-ACP synthase	1041	300	82.79	830	226.79	-1.45	6.88E-56	down	3261875	YP_201231.1	fabH	58582215
gi|58582216|ref| YP_201232.1|	XOO2593	3-oxoacyl-ACP reductase	855	125	42.00	304	101.14	-1.27	3.4E-17	down	3261876	YP_201232.1	fabG	58582216
gi|58582217|ref| YP_201233.1|	XOO2594	dehydrogenase	753	186	70.96	476	179.81	-1.34	8.7E-29	down	3261877	YP_201233.1		58582217
gi|58582219|ref| YP_201235.1|	XOO2596	ring hydroxylating dioxygenase subunit alpha	1146	384	96.26	848	210.48	-1.13	8.69E-39	down	3261879	YP_201235.1		58582219
gi|58582221|ref| YP_201237.1|	XOO2598	hypothetical protein	1515	497	94.24	1008	189.26	-1.01	3.16E-38	down	3261881	YP_201237.1		58582221
gi|58582223|ref| YP_201239.1|	XOO2600	flagellar protein	516	84	46.76	308	169.79	-1.86	6.85E-30	down	3261883	YP_201239.1	fliE	58582223
gi|58582224|ref| YP_201240.1|	XOO2601	flagellar MS-ring protein	1725	630	104.92	2431	400.87	-1.93	3.63E-242	down	3261884	YP_201240.1	fliF	58582224
gi|58582225|ref| YP_201241.1|	XOO2602	flagellar protein	1017	778	219.76	1850	517.43	-1.24	2.12E-96	down	3261885	YP_201241.1	fliG	58582225

gi|58582227|ref|YP_201243.1|	XOO2604	flagellar protein	1377	403	84.07	840	173.52	-1.05	5.06E-34	down	3261887	YP_201243.1	fliI	58582227
gi|58582230|ref|YP_201246.1|	XOO2607	flagellar protein	525	331	181.12	920	498.46	-1.46	2.85E-62	down	3261890	YP_201246.1	fliL	58582230
gi|58582231|ref|YP_201247.1|	XOO2608	flagellar motor switch protein FliM	1014	307	86.97	699	196.08	-1.17	4.31E-34	down	3261891	YP_201247.1	fliM	58582231
gi|58582232|ref|YP_201248.1|	XOO2609	flagellar protein	339	93	78.81	228	191.31	-1.28	2.71E-13	down	3261892	YP_201248.1	fliN	58582232
gi|58582233|ref|YP_201249.1|	XOO2610	flagellar protein	417	125	86.11	320	218.28	-1.34	1.23E-19	down	3261893	YP_201249.1	fliO	58582233
gi|58582234|ref|YP_201250.1|	XOO2611	flagellar biosynthesis protein FliP	846	209	70.97	446	149.96	-1.08	2.1E-19	down	3261894	YP_201250.1	fliP	58582234
gi|58582235|ref|YP_201251.1|	XOO2612	flagellar biosynthesis	270	52	55.33	208	219.13	-1.99	3.05E-22	down	3261895	YP_201251.1	fliQ	58582235
gi|58582236|ref|YP_201252.1|	XOO2613	flagellar biosynthetic protein	792	33	11.97	135	48.49	-2.02	4.89E-15	down	3261896	YP_201252.1	fliR	58582236
gi|58582237|ref|YP_201253.1|	XOO2614	diguanylate cyclase	2973	422	40.78	939	89.84	-1.14	1.62E-43	down	3261897	YP_201253.1		58582237
gi|58582239|ref|YP_201255.1|	XOO2616	diguanylate cyclase	2478	37	4.29	123	14.12	-1.72	2.95E-11	down	3261899	YP_201255.1		58582239
gi|161898983|ref|YP_201256.2|	XOO2617	flagellar biosynthesis protein FlhB	1131	98	24.89	504	126.76	-2.35	8.65E-65	down	3261900	YP_201256.2	flhB	161898983
gi|58582241|ref|YP_201257.1|	XOO2618	flagellar biosynthesis protein FlhA	2094	133	18.25	600	81.50	-2.16	1.49E-69	down	3261901	YP_201257.1	flhA	58582241
gi|122879200|ref|YP_201258.6|	XOO2619	flagellar biosynthesis regulator FlhF	1659	218	37.75	1184	203.01	-2.43	7.05E-157	down	3261902	YP_201258.6	flhF	122879200
gi|58582243|ref|YP_201259.1|	XOO2620	flagellar biosynthesis switch protein	960	61	18.25	598	177.19	-3.28	5.89E-108	down	3261903	YP_201259.1	fleN	58582243
gi|58582244|ref|YP_201260.1|	XOO2621	RNA polymerase sigma factor FliA	756	158	60.04	2112	794.65	-3.73	0	down	3261905	YP_201260.1	fliA	58582244
gi|58582245|ref|YP_201261.1|	XOO2622	chemotaxis protein	393	97	70.90	1134	820.77	-3.53	1.16E-216	down	3261904	YP_201261.1	cheY	58582245
gi|58582246|ref|YP_201262.1|	XOO2623	chemotaxis related protein	627	52	23.82	760	344.79	-3.86	6.96E-155	down	3261906	YP_201262.1	cheZ	58582246
gi|58582247|ref|YP_201263.1|	XOO2624	chemotaxis related protein	1659	213	36.88	2043	350.29	-3.25	0	down	3261907	YP_201263.1	cheA	58582247
gi|58582265|ref|YP_201281.1|	XOO2642	transporter	3501	166	13.62	80	6.50	1.07	1.04E-07	up	3261925	YP_201281.1	acrD	58582265
gi|58582271|ref|YP_201287.1|	XOO2648	transposase	1017	776	219.19	82	22.93	3.26	2.43E-140	up	3261935	YP_201287.1		58582271
gi|58582284|ref|YP_201300.1|	XOO2661	cytochrome D ubiquinol oxidase subunit II	258	42	46.76	13	14.33	1.71	0.0001987	up	3261948	YP_201300.1	cydB	58582284
gi|58582361|ref|YP_201377.1|	XOO2738	virulence protein	1263	127	28.89	267	60.13	-1.06	1.31E-11	down	3262026	YP_201377.1	acvB	58582361
gi|58582372|ref|YP_201388.1|	XOO2749	transposase	1017	1045	295.18	91	25.45	3.54	8.3E-202	up	3262038	YP_201388.1		58582372
gi|122879214|ref|YP_201421.6|	XOO2782	glutamine synthetase	1386	446	92.44	905	185.73	-1.01	1.94E-34	down	3265576	YP_201421.6	glnA	122879214
gi|58582410|ref|YP_201426.1|	XOO2787	transcriptional regulator	1299	50	11.06	1084	237.37	-4.42	2.32E-238	down	3264645	YP_201426.1	rrpX	58582410
gi|58582430|ref|YP_201446.1|	XOO2807	transposase	1008	751	214.03	78	22.01	3.28	9.5E-137	up	3265478	YP_201446.1		58582430
gi|58582453|ref|YP_201469.1|	XOO2830	flagellar motor protein	741	97	37.60	1023	392.70	-3.38	6.09E-189	down	3265596	YP_201469.1	motC	58582453
gi|58582454|ref|YP_201470.1|	XOO2831	flagellar motor protein MotD	861	41	13.68	474	156.59	-3.52	8.49E-91	down	3265626	YP_201470.1	motB	58582454
gi|58582455|ref|YP_201471.1|	XOO2832	chromosome partioning protein	783	50	18.34	713	259.02	-3.82	1.82E-144	down	3265627	YP_201471.1	parA	58582455
gi|58582456|ref|YP_201472.1|	XOO2833	chemotaxis protein	1023	140	39.31	1838	511.06	-3.70	0	down	3265624	YP_201472.1	cheW	58582456
gi|58582457|ref|YP_201473.1|	XOO2834	hypothetical protein	309	210	195.23	1342	1235.37	-2.66	6.45E-197	down	3265595	YP_201473.1		58582457
gi|58582458|ref|YP_201474.1|	XOO2835	chemotaxis response regulator	366	75	58.87	766	595.32	-3.34	4.09E-140	down	3265594	YP_201474.1	cheY	58582458
gi|58582459|ref|YP_201475.1|	XOO2836	chemotaxis protein	2010	1137	162.50	10270	1453.37	-3.16	0	down	3265621	YP_201475.1	cheA	58582459

gi|58582463|ref|YP_201479.1|	XOO2840	chemotaxis protein	2418	171	20.32	892	104.93	-2.37	3.74E-115	down	3265591	YP_201479.1	tsr	58582463
gi|58582464|ref|YP_201480.1|	XOO2841	transposase	1017	850	240.10	86	24.05	3.32	3.77E-156	up	3265619	YP_201480.1		58582464
gi|122879220|ref|YP_201481.6|	XOO2842	chemotaxis protein	2259	270	34.33	3730	469.67	-3.77	0	down	3265620	YP_201481.6	tsr	122879220
gi|58582467|ref|YP_201483.1|	XOO2844	chemotaxis protein	2313	448	55.64	8018	986.04	-4.15	0	down	3265615	YP_201483.1	tsr	58582467
gi|58582470|ref|YP_201486.1|	XOO2847	chemotaxis protein	2262	494	62.74	4646	584.24	-3.22	0	down	3265612	YP_201486.1	tsr	58582470
gi|122879221|ref|YP_201487.6|	XOO2848	chemotaxis protein	2262	460	58.42	2593	326.07	-2.48	0	down	3266135	YP_201487.6	tsr	122879221
gi|58582472|ref|YP_201488.1|	XOO2849	hypothetical protein	798	14	5.04	243	86.62	-4.10	4.38E-52	down	3265613	YP_201488.1		58582472
gi|58582473|ref|YP_201489.1|	XOO2850	chemotaxis protein	408	31	21.83	624	435.04	-4.32	5.29E-136	down	3265752	YP_201489.1	cheW	58582473
gi|58582480|ref|YP_201496.1|	XOO2857	chemotaxis protein methyltransferase	867	34	11.27	895	293.63	-4.70	4.7E-202	down	3265726	YP_201496.1	cheR	58582480
gi|58582506|ref|YP_201522.1|	XOO2883	transposase	1017	721	203.66	70	19.58	3.38	1.36E-134	up	3265671	YP_201522.1		58582506
gi|58582563|ref|YP_201579.1|	XOO2940	recombination regulator RecX	528	920	500.54	226	121.75	2.04	8.35E-100	up	3265072	YP_201579.1	recX	58582563
gi|58582564|ref|YP_201580.1|	XOO2941	recombinase A	1122	17272	4422.19	3797	962.61	2.20	0	up	3265073	YP_201580.1	recA	58582564
gi|58582604|ref|YP_201620.1|	XOO2981	ABC transporter ATP-binding protein	1785	73	11.75	25	3.98	1.56	2.74E-06	up	3263532	YP_201620.1		58582604
gi|58582623|ref|YP_201639.1|	XOO3000	cytochrome C	1428	41	8.25	14	2.79	1.56	0.0006001	up	3263528	YP_201639.1		58582623
gi|58582635|ref|YP_201651.1|	XOO3012	hypothetical protein	711	60	24.24	27	10.80	1.17	0.0009497	up	3264945	YP_201651.1		58582635
gi|58582718|ref|YP_201734.1|	XOO3095	transposase	411	293	204.79	32	22.15	3.21	7.11E-53	up	3263681	YP_201734.1		58582718
gi|58582720|ref|YP_201736.1|	XOO3097	transposase	1017	592	167.22	43	12.03	3.80	7.67E-121	up	3265268	YP_201736.1		58582720
gi|58582732|ref|YP_201748.1|	XOO3109	transposase	1017	845	238.68	73	20.42	3.55	9.3E-164	up	3265261	YP_201748.1		58582732
gi|122879242|ref|YP_201764.6|	XOO3125	peptidase	1572	234	42.76	106	19.18	1.16	7.51E-12	up	3265253	YP_201764.6		122879242
gi|58582749|ref|YP_201765.1|	XOO3126	hypothetical protein	333	15	12.94	86	73.46	-2.51	1.29E-12	down	3265250	YP_201765.1		58582749
gi|58582818|ref|YP_201834.1|	XOO3195	type IV pilin	456	280	176.39	71	44.29	1.99	4.32E-30	up	3263720	YP_201834.1	pilE1	58582818
gi|58582819|ref|YP_201835.1|	XOO3196	protein PilY1	3525	3599	293.30	500	40.35	2.86	0	up	3265185	YP_201835.1	pilY1	58582819
gi|58582820|ref|YP_201836.1|	XOO3197	transposase	1089	2015	531.54	320	83.58	2.67	4.6E-299	up	3263722	YP_201836.1		58582820
gi|58582822|ref|YP_201838.1|	XOO3199	protein PilX	510	355	199.96	1024	571.13	-1.51	5.92E-73	down	3265171	YP_201838.1	pilX	58582822
gi|58582823|ref|YP_201839.1|	XOO3200	hypothetical protein	1167	776	191.02	3334	812.64	-2.09	0	down	3263719	YP_201839.1		58582823
gi|58582824|ref|YP_201840.1|	XOO3201	pre-pilin leader sequence	474	212	128.48	881	528.69	-2.04	9.36E-95	down	3265184	YP_201840.1	pilV	58582824
gi|58582825|ref|YP_201841.1|	XOO3202	pre-pilin like leader sequence	516	235	130.83	1102	607.48	-2.22	4.39E-131	down	3265169	YP_201841.1	fimT	58582825
gi|58582830|ref|YP_201846.1|	XOO3207	hypothetical protein	321	48	42.96	101	89.50	-1.06	0.0000604	down	3263715	YP_201846.1		58582830
gi|58582869|ref|YP_201885.1|	XOO3246	phosphodiesterase	2367	651	79.01	1466	176.17	-1.16	4.54E-69	down	3263846	YP_201885.1		58582869
gi|58582870|ref|YP_201886.1|	XOO3247	hypothetical protein	681	1046	441.24	2308	964.03	-1.13	6.39E-104	down	3265114	YP_201886.1		58582870
gi|58582891|ref|YP_201907.1|	XOO3268	hypothetical protein	480	34	20.35	81	48.00	-1.24	0.0000457	down	3265077	YP_201907.1		58582891
gi|58582907|ref|YP_201923.1|	XOO3284	IS1595 transposase	963	332	99.04	62	18.31	2.44	3.1E-45	up	3264177	YP_201923.1		58582907
gi|58582923|ref|YP_201939.1|	XOO3300	extracellular protease	1224	10266	2409.39	2347	545.42	2.14	0	up	3264167	YP_201939.1		58582923
gi|58582968|ref|YP_201984.1|	XOO3345	hypothetical protein	528	197	107.18	582	313.54	-1.55	3.19E-43	down	3264231	YP_201984.1		58582968
gi|58582969|ref|YP_201985.1|	XOO3346	transposase	957	549	164.80	48	14.27	3.53	2.38E-106	up	3264230	YP_201985.1		58582969
gi|58582976|ref|YP_201992.1|	XOO3353	transposase	357	68	54.72	13	10.36	2.40	8.39E-10	up	3264235	YP_201992.1		58582976

gi|58582977|ref|YP_201993.1|	XOO3354	transposase	303	178	168.76	32	30.04	2.49	2.9E-25	up	3264240	YP_201993.1		58582977
gi|58582987|ref|YP_202003.1|	XOO3364	hypothetical protein	453	88	55.80	313	196.54	-1.82	2.03E-29	down	3264284	YP_202003.1		58582987
gi|58582998|ref|YP_202014.1|	XOO3375	hypothetical protein	525	68	37.21	197	106.74	-1.52	8.15E-15	down	3264220	YP_202014.1		58582998
gi|122879253|ref|YP_202019.6|	XOO3380	fumarylacetoacetate hydrolase	714	229	92.14	1	0.40	7.85	1.52E-49	up	3264223	YP_202019.6		122879253
gi|58583004|ref|YP_202020.1|	XOO3381	large-conductance mechanosensitive channel	435	992	655.10	6	3.92	7.38	4.47E-220	up	3264222	YP_202020.1	mscL	58583004
gi|58583011|ref|YP_202027.1|	XOO3388	tRNA/rRNA methyltransferase	1062	230	62.21	1	0.27	7.86	9.93E-50	up	3264192	YP_202027.1		58583011
gi|58583014|ref|YP_202030.1|	XOO3391	transposase	1017	945	266.93	61	17.06	3.97	7.16E-198	up	3264196	YP_202030.1		58583014
gi|58583015|ref|YP_202031.1|	XOO3392	hypothetical protein	453	31	19.66	0	0.00	5.97	3.5E-08	up	3264195	YP_202031.1		58583015
gi|58583016|ref|YP_202032.1|	XOO3393	aminopeptidase	2727	264	27.81	0	0.00	9.06	4.92E-50	up	3264194	YP_202032.1	pepN	58583016
gi|58583017|ref|YP_202033.1|	XOO3394	acriflavin resistance protein	3126	307	28.21	1	0.09	8.28	1.83E-63	up	3263468	YP_202033.1	acrF	58583017
gi|58583018|ref|YP_202034.1|	XOO3395	RND efflux membrane fusion protein	957	132	39.62	1	0.30	7.06	1.02E-30	up	3264317	YP_202034.1		58583018
gi|58583019|ref|YP_202035.1|	XOO3396	bifunctional sulfate adenylyltransferase subunit 1/adenylylsulfate kinase	1980	445	64.56	1	0.14	8.81	2.7E-86	up	3266096	YP_202035.1	nodQ	58583019
gi|58583020|ref|YP_202036.1|	XOO3397	sulfate adenylyltransferase subunit 2	909	240	75.85	3	0.94	6.34	4.71E-57	up	3265783	YP_202036.1	raxP	58583020
gi|122879254|ref|YP_202039.6|	XOO3400	sulfite reductase subunit beta	1707	92	15.48	0	0.00	7.54	5.97E-21	up	3265787	YP_202039.6	cysI	122879254
gi|58583024|ref|YP_202040.1|	XOO3401	phosphoadenosine phosphosulfate reductase	726	42	16.62	0	0.00	6.41	1.15E-10	up	3265782	YP_202040.1	cysH	58583024
gi|58583235|ref|YP_202251.1|	XOO3612	hypothetical protein	1230	79	18.45	659	152.40	-3.05	2.83E-111	down	3264632	YP_202251.1		58583235
gi|58583269|ref|YP_202285.1|	XOO3646	alkyl hydroperoxide reductase	1647	598	104.30	1449	250.25	-1.26	2.84E-78	down	3264476	YP_202285.1	ahpF	58583269
gi|58583270|ref|YP_202286.1|	XOO3647	oxidative stress transcriptional regulator	942	248	75.63	803	242.48	-1.68	1.26E-66	down	3264456	YP_202286.1	oxyR	58583270
gi|58583348|ref|YP_202364.1|	XOO3725	hypothetical protein	723	543	215.75	268	105.44	1.03	4.38E-22	up	3264825	YP_202364.1		58583348
gi|58583360|ref|YP_202376.1|	XOO3737	transposase	1017	747	211.00	67	18.74	3.49	3.59E-143	up	3264813	YP_202376.1		58583360
gi|58583374|ref|YP_202390.1|	XOO3751	dihydrofolate reductase type III	495	338	196.15	133	76.43	1.36	2.66E-21	up	3264797	YP_202390.1	folA	58583374
gi|58583469|ref|YP_202485.1|	XOO3846	potassium-transporting ATPase subunit A	1794	68	10.89	27	4.28	1.35	0.0000631	up	3262419	YP_202485.1	kdpA	58583469
gi|58583533|ref|YP_202549.1|	XOO3910	transposase	1017	1041	294.05	100	27.97	3.39	1.31E-194	up	3266039	YP_202549.1		58583533
gi|58583558|ref|YP_202574.1|	XOO3935	two-component system regulatory protein	441	158	102.92	425	274.13	-1.41	7.35E-28	down	3262269	YP_202574.1		58583558
gi|58583611|ref|YP_202627.1|	XOO3988	response regulator	966	16	4.76	611	179.91	-5.24	1.95E-142	down	3262481	YP_202627.1		58583611
gi|58583612|ref|YP_202628.1|	XOO3989	aminopeptidase	2073	12454	1725.83	3911	536.65	1.69	0	up	3262437	YP_202628.1	pepN	58583612
gi|58583626|ref|YP_202642.1|	XOO4003	hypothetical protein	339	152	128.80	16	13.43	3.26	3.1E-28	up	3262458	YP_202642.1		58583626
gi|58583631|ref|YP_202647.1|	XOO4008	two-component system regulatory protein	738	601	233.94	1233	475.24	-1.02	8.12E-48	down	3262463	YP_202647.1	algR	58583631
gi|58583644|ref|YP_202660.1|	XOO4021	histidine kinase-response regulator hybrid protein	2814	447	45.63	1455	147.08	-1.69	8.87E-121	down	3262476	YP_202660.1		58583644
gi|122879312|ref|YP_202669.6|	XOO4030	trehalase	1674	213	36.55	633	107.56	-1.56	2.64E-47	down	3262384	YP_202669.6	treA	122879312

gi|58583671|ref|YP_202687.1|	XOO4048	threonine aldolase	909	99	31.29	44	13.77	1.18	0.0000108	up	3262409	YP_202687.1		58583671
gi|122879319|ref|YP_202715.6|	XOO4076	nuclease	888	67	21.67	16	5.13	2.08	2.94E-08	up	3262370	YP_202715.6	nucH	122879319
gi|58583700|ref|YP_202716.1|	XOO4077	Pyruvate dehydrogenase e1 component subunit alpha	669	39	16.75	11	4.68	1.84	0.0001548	up	3262371	YP_202716.1	pdhA	58583700
gi|58583702|ref|YP_202718.1|	XOO4079	transposase	912	877	276.24	90	28.07	3.30	3.1E-160	up	3262285	YP_202718.1		58583702
gi|58583704|ref|YP_202720.1|	XOO4081	Pyruvate dehydrogenase E1 component subunit beta	510	235	132.37	49	27.33	2.28	7.46E-30	up	3262287	YP_202720.1	pdhB	58583704
gi|58583705|ref|YP_202721.1|	XOO4082	hypothetical protein	381	25	18.85	3	2.24	3.07	0.0000389	up	3262288	YP_202721.1		58583705
gi|58583720|ref|YP_202736.1|	XOO4097	hypothetical protein	378	30	22.80	72	54.18	-1.25	0.0001173	down	3262303	YP_202736.1		58583720
gi|58583728|ref|YP_202744.1|	XOO4105	transposase	1104	2423	630.48	665	171.34	1.88	1.1E-234	up	3262311	YP_202744.1		58583728
gi|58583735|ref|YP_202751.1|	XOO4112	hypothetical protein	282	54	55.01	22	22.19	1.31	0.0005677	up	3262318	YP_202751.1		58583735
gi|58583740|ref|YP_202756.1|	XOO4117	transposase	1002	1744	500.00	319	90.56	2.47	3.99E-237	up	3262323	YP_202756.1		58583740
gi|58583741|ref|YP_202757.1|	XOO4118	transposase	609	177	83.49	63	29.43	1.50	2.52E-13	up	3262324	YP_202757.1		58583741
gi|58583744|ref|YP_202760.1|	XOO4121	transposase	1017	1093	308.74	97	27.13	3.51	9.08E-210	up	3262327	YP_202760.1		58583744
gi|58583770|ref|YP_202786.1|	XOO4147	transposase	834	630	217.00	61	20.80	3.38	7.73E-118	up	3261735	YP_202786.1		58583770
gi|58583773|ref|YP_202789.1|	XOO4150	bacterioferritin-associated ferredoxin	270	33	35.11	78	82.17	-1.23	0.0000746	down	3261752	YP_202789.1		58583773
gi|58583816|ref|YP_202832.1|	XOO4193	transposase	1017	785	221.74	71	19.86	3.48	5.41E-150	up	3263379	YP_202832.1		58583816
gi|58583827|ref|YP_202843.1|	XOO4204	hypothetical protein	612	147	69.00	65	30.21	1.19	4.17E-08	up	3263154	YP_202843.1		58583827
gi|58583840|ref|YP_202856.1|	XOO4217	hypothetical protein	159	68	122.86	32	57.25	1.10	0.0007627	up	3263171	YP_202856.1		58583840
gi|58583843|ref|YP_202859.1|	XOO4220	hypothetical protein	1335	119	25.61	549	116.98	-2.19	6.75E-65	down	3263174	YP_202859.1		58583843
gi|58583846|ref|YP_202862.1|	XOO4223	transposase	135	18	38.30	0	0.00	5.18	0.0000449	up	3263177	YP_202862.1		58583846
gi|58583847|ref|YP_202863.1|	XOO4224	transposase	834	725	249.72	73	24.90	3.33	1.39E-133	up	3263178	YP_202863.1		58583847
gi|58583898|ref|YP_202914.1|	XOO4275	hypothetical protein	519	78	43.17	39	21.37	1.01	0.0007525	up	3263232	YP_202914.1		58583898
gi|58583904|ref|YP_202920.1|	XOO4281	transposase	609	165	77.83	27	12.61	2.63	7.37E-25	up	3263239	YP_202920.1		58583904
gi|122879337|ref|YP_202926.6|	XOO4287	hypothetical protein	2109	386	52.58	787	106.15	-1.01	2.15E-30	down	3263245	YP_202926.6		122879337
gi|58583956|ref|YP_202972.1|	XOO4333	5-methyltetrahydropteroyltriglutamate/homocysteine S-methyltransferase	1029	1668	465.66	6340	1752.57	-1.91	0	down	3263294	YP_202972.1	metE	58583956
gi|58583957|ref|YP_202973.1|	XOO4334	hypothetical protein	1167	1723	424.13	6151	1499.26	-1.82	0	down	3263295	YP_202973.1		58583957
gi|58583958|ref|YP_202974.1|	XOO4335	NADH-dependent FMN reductase	603	505	240.58	1451	684.47	-1.51	1.81E-102	down	3263296	YP_202974.1	sflA	58583958
gi|58583979|ref|YP_202995.1|	XOO4356	alpha/beta hydrolase	315	84	76.60	23	20.77	1.88	5.44E-09	up	3263317	YP_202995.1		58583979
gi|58583980|ref|YP_202996.1|	XOO4357	transposase	1017	718	202.81	90	25.17	3.01	5.54E-121	up	3263318	YP_202996.1		58583980
gi|58584054|ref|YP_203070.1|	XOO4431	TonB-dependent receptor	2970	111	10.74	269	25.76	-1.26	3.15E-15	down	3262990	YP_203070.1	cirA	58584054
gi|58584063|ref|YP_203079.1|	XOO4440	transposase	561	652	333.87	46	23.32	3.84	5.67E-134	up	3262999	YP_203079.1		58584063
gi|58584064|ref|YP_203080.1|	XOO4441	transposase	450	135	86.18	20	12.64	2.77	1.24E-21	up	3263000	YP_203080.1		58584064
gi|58584065|ref|YP_203081.1|	XOO4442	hypothetical protein	390	32	23.57	6	4.38	2.43	0.000039	up	3263001	YP_203081.1		58584065

gi|58584095|ref|YP_203111.1|	XOO4472	hypothetical protein	2655	234	25.32	618	66.21	-1.39	5.17E-39	down	3263033	YP_203111.1	58584095
gi|58584102|ref|YP_203118.1|	XOO4479	transposase	1026	904	253.11	94	26.06	3.28	2.58E-164	up	3263040	YP_203118.1	58584102
gi|58584143|ref|YP_203159.1|	XOO4520	transposase	690	879	365.95	90	37.10	3.30	9.96E-161	up	3262907	YP_203159.1	58584143
gi|58584194|ref|YP_203210.1|	XOO4571	transposase	1059	1029	279.13	110	29.55	3.24	5.97E-185	up	3262828	YP_203210.1	58584194
gi|58584203|ref|YP_203219.1|	XOO4580	hypothetical protein	1128	110	28.01	24	6.05	2.21	5.86E-14	up	3262837	YP_203219.1	58584203
gi|58584237|ref|YP_203253.1|	XOO4614	hypothetical protein	510	567	319.37	251	139.99	1.19	1.01E-28	up	3262754	YP_203253.1	58584237
gi|58584238|ref|YP_203254.1|	XOO4615	hypothetical protein	444	6082	3935.06	2455	1572.79	1.32	0	up	3262778	YP_203254.1	58584238
gi|58584239|ref|YP_203255.1|	XOO4616	IS1478 transposase	687	318	132.97	875	362.29	-1.45	2.11E-58	down	3262779	YP_203255.1	58584239
gi|121632018|ref|YP_001001379.1|	XOO4703	transposase	285	60	60.48	3	2.99	4.34	5.1E-14	up	4699396	YP_001001379.1	121632018
gi|121632044|ref|YP_001001405.1|	XOO4734	transposase	561	534	273.44	50	25.35	3.43	3.42E-101	up	4699423	YP_001001405.1	121632044
gi|121632082|ref|YP_001001444.1|	XOO4779	hypothetical protein	375	2313	1771.87	24	18.20	6.60	0	up	4699461	YP_001001444.1	121632082
gi|121632084|ref|YP_001001446.1|	XOO4782	transposase	168	279	477.07	108	182.86	1.38	3.52E-18	up	4699463	YP_001001446.1	121632084
gi|121632089|ref|YP_001001451.1|	XOO4790	hypothetical protein	234	59	72.43	16	19.45	1.90	1.25E-06	up	4699468	YP_001001451.1	121632089
gi|121632106|ref|YP_001001468.1|	XOO4825	hypothetical protein	714	343	138.00	696	277.28	-1.01	1.09E-26	down	4699484	YP_001001468.1	121632106
gi|121632112|ref|YP_001001475.1|	XOO4876	transposase	168	195	333.44	97	164.23	1.02	2.35E-08	up	4699489	YP_001001475.1	121632112

Table S2. DEGs between WT and Δ*dgcA* mutant.

Names	Sequences
Xoo3988-in-F	GCTGGCGGTGACGTTTATTG
Xoo3988-in-R	CGCATGTTTCTTGAGGTCGC
Xoo3989-in-F	GGACGATGCGTACCACTTCA
Xoo3989-in-R	CGGCTTGGCTTTTTCGAAGG
Xoo3988-3989-F	CCACGCTCAAGCTCAATCAA
Xoo3988-3989-R	CGACTGGCTGAACATGAAAGG
dgcA-up-F	CGAATTCTCACCGATAAAGATGCCG
dgcA-up-R	GAGGATCCGGCGTACAAGCGCAT
dgcA-down-F	CCCGGTCGACCATCAGGTGACCT
dgcA-down-R	GTCAAGCTTACCGGCGCCAAGGC
Gm-F	GCGGATCCCCAGTTGACATAAGC
Gm-R	GAACGTCGACTTAGGTGGCGGTAC
dgcA-F2	AGGGATCCATGCGCTTGTACGCCGGAC
dgcA-R2	GGATCCTCAGTGGTGGTGGTGGTGGTGGGTCACCTGATGACCGCGCG
M13-F	TGTAAAACGACGGCCAGT
M13-R	CAGGAAACAGCTATGACC
Kan-F	ATGATTGAACAAGATGGATTGCACG
Kan-R	TCAGAAGAACTCGTCAAGAAGGC

Table S3. Key primers used in this study. Forward (F) and reverse (R) primers are listed from 5’ to 3’. The restriction sites are underlined.
